# Omega-3, omega-6 and total dietary polyunsaturated fat on cancer incidence: systematic review and meta-analysis of randomised trials

**DOI:** 10.1038/s41416-020-0761-6

**Published:** 2020-02-29

**Authors:** Sarah Hanson, Gabrielle Thorpe, Lauren Winstanley, Asmaa S. Abdelhamid, Lee Hooper, on behalf of the PUFAH group, Asmaa Abdelhamid, Sarah Ajabnoor, Faye Alabdulghafoor, Lena Alkhudairy, Priti Biswas, Julii Brainard, Charlene Bridges, Tracey J Brown, Katherine Deane, Daisy Donaldson, Sarah Hanson, Lee Hooper, Oluseyi Florence Jimoh, Nicole Martin, Alex O’Brien, Karen Rees, Lena Alkhudairy, Fujian Song, Gabrielle Thorpe, Xia Wang, Lauren Winstanley

**Affiliations:** 1 School of Health Sciences, University of East Anglia, Norwich, NR4 7TJ UK; 2 Norwich Medical School, University of East Anglia, Norwich, NR4 7TJ UK; 3 Warwick Medical School, University of Warwick, Coventry, CV4 7AL UK; 4 Health Sciences, University of East Anglia, Norwich, NR4 7TJ UK; 5 Cochrane Heart Group, University College London, London, WC1E 6BT UK

**Keywords:** Risk factors, Oncology

## Abstract

**Background:**

The relationship between long-chain omega-3 (LCn3), alpha-linolenic acid (ALA), omega-6 and total polyunsaturated fatty acid (PUFA) intakes and cancer risk is unclear.

**Methods:**

We searched Medline, Embase, CENTRAL and trials registries for RCTs comparing higher with lower LCn3, ALA, omega-6 and/or total PUFA, that assessed cancers over ≥12 months. Random-effects meta-analyses, sensitivity analyses, subgrouping, risk of bias and GRADE were used.

**Results:**

We included 47 RCTs (108,194 participants). Increasing LCn3 has little or no effect on cancer diagnosis (RR1.02, 95% CI 0.98–1.07), cancer death (RR0.97, 95% CI 0.90–1.06) or breast cancer diagnosis (RR1.03, 95% CI 0.89–1.20); increasing ALA has little or no effect on cancer death (all high/moderate-quality evidence). Increasing LCn3 (NNTH 334, RR1.10, 95% CI 0.97–1.24) and ALA (NNTH 334, RR1.30, 95% CI 0.72–2.32) may slightly increase prostate cancer risk; increasing total PUFA may slightly increase risk of cancer diagnosis (NNTH 125, RR1.19, 95% CI 0.99–1.42) and cancer death (NNTH 500, RR1.10, 95% CI 0.48–2.49) but total PUFA doses were very high in some trials.

**Conclusions:**

The most extensive systematic review to assess the effects of increasing PUFAs on cancer risk found increasing total PUFA may very slightly increase cancer risk, offset by small protective effects on cardiovascular diseases.

## Background

Cancer is a leading cause of morbidity and mortality worldwide with ~17 million new cases and 9.6 million cancer-related deaths in 2018.^1^ The most common cancers worldwide are lung, female breast, bowel and prostate cancer, accounting for 40% of cancers diagnosed.^1^ 23% of UK breast cancer cases are thought to be preventable, with causes including overweight and obesity (8%), alcohol (8%), not breastfeeding (5%), post-menopausal hormones (2%) and oral contraceptives (<1%).^1^ Preventability appears to vary so 79% of lung cancer cases are preventable (and mainly due to smoking), 54% of bowel cancer (causes including too little dietary fibre, processed meat, overweight and obesity, alcohol, smoking and sedentary behaviour) and an unknown proportion of prostate cancer (risk factors are unclear).^1^ Every sixth death in the world is due to cancer^2^ and in the USA cancer expenditure is projected as $156 billion by 2020,^3^ so even small beneficial or harmful effects could be important. The other major health risk worldwide is cardiovascular disease, responsible for 37% of premature deaths due to non-communicable disease in 2012, where cancers were responsible for 27%.^4^

Dietary polyunsaturated fatty acids (PUFA) have been postulated as a modifiable component of lifestyle that could influence cancer risk. PUFA includes long-chain omega-3 (LCn3 including eicosapentaenoic acid and docosapentaenoic acid), alpha-linolenic acid (ALA, a shorter chain omega-3) and omega-6 fats (including linoleic acid, LA). Polyunsaturated fats are common healthy eating choices, and fish oil (LCn3) and flaxseed (ALA) supplements commonly consumed.^5^ Potential mechanisms for PUFAs in cancer aetiology include their being precursors to lipid mediators regulating metabolic pathways and inflammatory responses,^6^ oxidative stress, and changes in membrane composition that could affect cell signalling pathways.^7^ Reducing dietary fat (including PUFAs) appears to result in lower weight in adults,^8^ so lower PUFA intake (as part of general fat reduction) could offer protective effects against those cancers that are associated with overweight. These mechanisms suggest that omega-3 may be protective, and omega-6 and total PUFA may exacerbate cancer risk. However, oily fish and fish oil capsules may contain contaminants such as mercury and dioxins, potential carcinogens.^9–12^

Evidence for effects of polyunsaturated fats on risk of cancer is conflicting. An early RCT, the Lyon Diet Heart Study, suggested that a Mediterranean type diet, supplemented with an experimental canola (rapeseed) oil-based margarine rich in oleic and ALA, reduced cancer diagnoses by 61% compared to those on the American Heart Association diet.^13^ Within the Japanese population, whose traditional diet is rich in oily fish, incidence of some cancers has increased with more westernised food consumption and lifestyles.^6^ One systematic review of cohort studies did not pool data but found some cohorts with positive associations, some with negative associations and more with null associations for omega-3 and a variety of cancers, including breast and prostate cancer – overall there was no trend to suggest that omega-3 fatty acids are associated with total cancer risk.^14^ A systematic review of 10 RCTs comparing high to low omega-3 intake for at least 6 months found no evidence that increasing omega-3 fats altered cancer incidence.^15^ Later meta-analysis of RCTs increasing omega 3 intake over at least 6 months found omega-3 supplementation increased the risk of cancer by 10% but this was not statistically significant,^16^ and this review did not analyse for specific cancer types, provided limited information on dosage and did not stratify by supplementation level.

The Mediterranean diet, which is high in polyunsaturated fats, has attracted attention because of the historically lower breast cancer rates in Mediterranean countries than in other parts of Europe and the United States.^17,18^ A cohort study of over 35,000 post-menopausal US women suggested that taking omega-3 supplements was associated with a 32% reduction in breast cancer risk,^19^ although other cohort studies are not consistent in this relationship.^20^ A large European cohort study (EPIC) found no association between fatty fish consumption and breast cancer risk.^21^ Comprehensive systematic reviews of observational studies suggested no relationship between total polyunsaturated fat intake and risk of breast cancer^22^ or omega-3 intake and breast cancer risk.^23^

Two nested case-control studies of men suggested that high serum long-chain omega-3 fatty acids were associated with increased risk of prostate cancer and high-grade prostate cancer,^24,25^ but a systematic review found inadequate data to determine whether fish-derived omega-3 fatty acids were associated with prostate cancer incidence and progression.^26^

Some polyunsaturated fats are essential in the human diet, and UK dietary reference values suggest we need to eat at least 6.5% of our energy intake in the form of cis-polyunsaturated fats.^27^ Further increasing polyunsaturated fat intake is associated with healthy eating and prevention of cardiovascular disease in the general public, but is still scientifically controversial.^28^ The use of supplements as additions or replacements to food stuff has gained traction with the general public. It has been estimated that approximately 38% of American adults use complementary medicines and fish oil, omega 3 or DHA supplements are the most commonly used non-vitamin, non-mineral natural product (37.4%) and flaxseed the 4th (15.9%).^5^ LCn3 is ingested in the form of oily fish or fish oil (often fish liver oil) capsules, however, these may contain contaminants. Heavy metals such as mercury, cadmium, chromium, nickel, lead and cobalt and toxic compounds such as dioxins have been found in fish and fish oils representing a potential risk to health.^9–12^ It is therefore important to assess both potential benefits and harms of increasing omega-3, omega-6 and total polyunsaturated fats on cancer risk to better inform members of the public considering dietary change or supplementation.

As previous systematic reviews of trials and observational studies have been equivocal about effects of omega-3, omega-6 and total PUFA on total, breast and prostate cancer risk,^15,16,22,23,26,29–32^ this review assessed the risks and protective effects of increasing omega 3, omega 6 and total polyunsaturated fat (PUFA) intake on total, breast and prostate cancer incidence in adults, gathering a much larger set of randomised trials than has previously been assessed as it included trials where cancer diagnosis was not the primary outcome, but cancer diagnosis or mortality data were available. As this systematic review was conducted as part of a series of systematic reviews assessing a range of health effects of omega-3, omega-6 and total PUFA^33–38^ (Ajabnoor et al., personal communication, Brainard et al., personal communication) we have been able to compare health benefits and harms across the major causes of mortality and morbidity in developed countries: cancer and cardiovascular disease.

## Methods

Methods for the series have been reported in detail (including the PRISMA flow diagram and detailed search strategies).^39^ This review’s protocol was registered on PROSPERO^40^ and its specific methods are summarised below.

### Inclusion criteria

We included randomised controlled trials (RCTs) that compared higher versus lower LCn3, ALA, omega-6 and/or total PUFA in adults aged at least 18 years, who were not pregnant or seriously ill. Participants could be free of cancer, at increased risk of cancer or with a previous cancer diagnosis, but were excluded if they were currently undergoing cancer treatment. The minimum study duration was 1 year (≥52 weeks) reflecting metabolic studies suggesting 6 months is the minimum supplementation duration required to equilibrate LCn3 into most body compartments,^41^ plus a further 6 months to influence cancer development.

Interventions could consist of foods, oral supplements (oil, capsules, or enriched foods) or advice, to increase or decrease omega-3, omega-6 and/or total PUFA intake, or achieve a change of ≥10% of baseline intake, comparing higher versus lower PUFA intake. Studies were excluded if they examined lifestyle or dietary interventions in addition to PUFA unless effects of the PUFA could be separated out.

Primary outcomes included:

- New diagnosis of breast cancer
- Breast cancer mortality
- New diagnosis of any cancer
- Any cancer mortality

Secondary outcomes included prostate cancer diagnosis and mortality (added post-hoc to complement prostate-specific antigen (PSA) data), markers of cancer risk (including breast density and PSA), body weight and measures of adiposity, quality of life, and dropouts.

### Methods for identification of studies

We searched Cochrane CENTRAL, Medline and Embase to 27 April 2017, ClinicalTrials.com and WHO International Clinical Trials Registry Platform to September 2016 and reassessed all ongoing trials in December 2018. We checked included trials of relevant systematic reviews, and wrote to authors of included studies for additional trial data, creating a database of trials that randomised participants to increased omega-3, omega-6 or total PUFA compared to lower omega-3, omega-6 or total PUFA.^39^ From this database, trials with duration of at least 12 months and data collected on any primary outcome were included in this review, even if study objectives were not primarily to assess effects on cancer, or those outcomes were not published.

Study inclusion, data extraction and risk of bias assessment (onto a specially developed form) were conducted independently in duplicate. We assessed Cochrane risk of bias domains^42^ plus risk from compliance problems and attention bias.^39^ We considered supplementation trials to be at low summary risk of bias where randomisation, allocation concealment, blinding of participants, personnel and outcome assessors were judged adequate (all other trials were considered at moderate or high risk of bias). Dietary advice trials were at low summary risk of bias where randomisation, allocation concealment and blinding of outcome assessors were assessed adequate.^39^

### Data synthesis

Primary analyses assessed effects of total PUFA, omega-6, LCn3 and ALA using random-effects Mantel–Haenszel meta-analysis (as dietary interventions are heterogeneous by their nature^43^) in Review Manager 5.3.^44^ Treatment/control differences in outcomes were combined across studies using risk ratios (RR) or mean differences (MD), the at-risk population included only men for prostate cancer and women for breast cancer. Change from baseline in each arm with standard deviations were used for continuous outcomes where available, otherwise endpoint data were used.^43^ Pre-specified sensitivity analyses included fixed effects analysis, limiting analysis to studies at low summary risk of bias, and limiting to trials randomising ≥100 participants. At the request of our funders, we added sensitivity analyses limiting to studies at low risk for compliance issues. At the request of referees, we added sensitivity analyses using Peto fixed-effects analysis (creating odds ratios), to ensure that our findings are robust to analysis methods despite the inclusion of trials with rare events. Pre-specified subgroup analyses were conducted for outcomes with ≥8 studies by intervention type, replacement, dose, duration, age, sex and cancer risk (normal cancer risk/ increased risk/ previous cancer).^39^ We planned to sub-group also by medications used, baseline omega-3, omega-6 or total PUFA intake, pre- or post-menopausal, BMI, ethnicity and omega-3/omega-6 ratio, however, this information was not available in most trials, so subgrouping was not attempted. Heterogeneity was assessed using I^2^ and considered important where >50%.^45^ Small study bias was assessed using funnel plots where at least 10 trials were included in a meta-analysis.^46^ Data from individual participants were only counted once in any meta-analytical pooling.

Effect sizes were interpreted as agreed with the WHO Nutrition Guidance Expert Advisory Group (NUGAG) Subgroup on Diet and Health who commissioned this research.^39^ A risk ratio less than 0.92 or greater than 1.08 was considered a potentially relevant clinical effect (RR 0.92–1.08 was considered “little or no effect”), while a mean difference between arms of at least 10% of baseline was required for a relevant clinical effect for markers. Where we found a suggested effect we quantified the effect using number needed to treat for an additional benefit (NNTB) or number needed to treat to cause an additional harm (NNTH).^47^ Outcome data were interpreted using GRADE assessment, drafted by LH then discussed and agreed with WHO NUGAG.^39^ Where sensitivity analyses using Mantel–Haenszel or Peto fixed-effects analyses were not consistent with the main random-effects analysis we downgraded (for inconsistency), and where sensitivity analyses including only trials at low summary risk of bias, or only trials with good compliance differed from the main analysis we downgraded (for risk of bias). Where GRADE suggested data of very low-quality we did not interpret effect sizes. Where data were of low-quality, we used the term “may”, moderate-quality evidence warranted “probably” in describing effects. Summary of findings’ (GRADE) tables show effects on all cancers, breast cancer and prostate cancer diagnoses and deaths (marker evidence strengthened or weakened findings for relevant cancers).

## Results

We included 47 RCTs (49 comparisons). Thirty-four trials (97,548 participants) assessed effects of LCn3, three (3179 participants) assessed ALA, eight (4976 participants) assessed omega-6 and 9 trials (11,573 participants) assessed total PUFA (Supplementary Fig. 1 and Supplementary Table 1). As several trials assessed multiple PUFA interventions, numbers are not additive. Thirty-eight trials included participants with normal baseline cancer risk, three with cancer risk factors and six trials with previously diagnosed cancer. Most trials provided supplementary capsules, but omega-6 and total PUFA trials often provided dietary advice and/or supplementary foods (enriched margarines or nuts), and one institutional trial provided all food. Mean trial duration was >30 months and trials were conducted in Europe (20 trials), North America (15), Japan (5), Australia/ New Zealand (2), or over several continents (5). Seventeen RCTs were assessed as being at low summary risk of bias (Supplementary Fig. 2, Supplementary Table 1).

Results are discussed briefly here, fuller results are presented in the supplementary materials (Supplementary Figs. 3–5 are funnel plots relating to effects of LCn3, Supplementary Figs. 6–9 are forest plots depicting effects of omega-3, omega-6 and total PUFA on cancer-related outcomes, Supplementary Fig. 10 the funnel plot for effects of total PUFA on cancer diagnosis, Supplementary Figs. 11–15 further forest plots, Supplementary Tables 2–6 detail results of all meta-analyses and GRADE table on effects of omega-3, Supplementary Tables 7–9 detail meta-analyses and the GRADE table for omega-6, Supplementary Tables 10–12 are meta-analyses and GRADE table for total PUFA).

### Effects of increasing long-chain omega-3

**Fig. 1:**
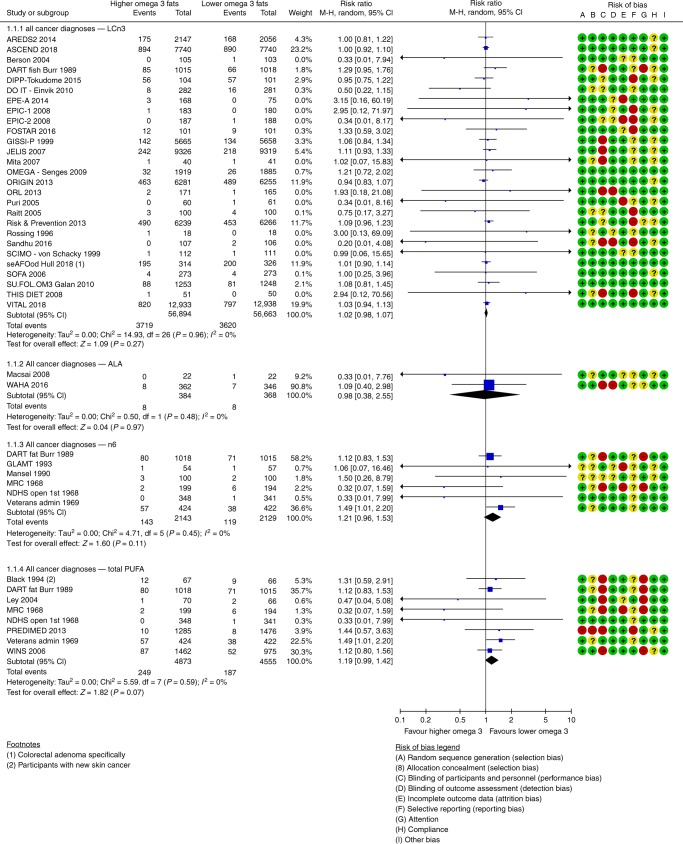
Forest plot showing effects of increasing omega-3, omega-6 and total PUFA on any cancer diagnosis, using random-effects meta-analyses.

**Fig. 2:**
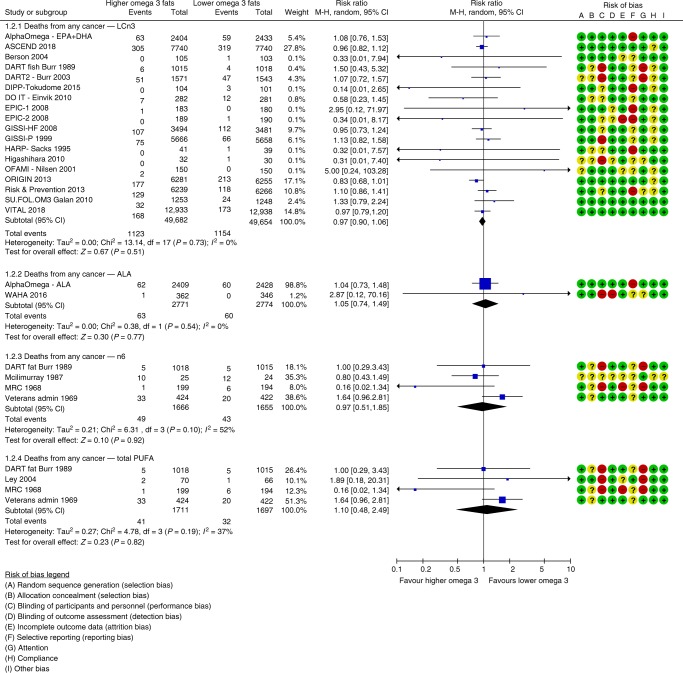
Forest plot showing effects of increasing omega-3, omega-6 and total PUFA on death from any cancer, using random-effects meta-analyses.

Increasing LCn3 has little or no effect on risk of diagnosis of any cancer (high-quality evidence) and probably has little or no effect on risk of cancer death (moderate-quality evidence). We meta-analysed 27 trials (113,557 participants, 7339 diagnoses, mean duration 32 months, mean dose 1.7 g/d LCn3) assessing effects of LCn3 on cancer diagnosis (RR 1.02, 95% CI 0.98 to 1.07, I^2^ 0%, Fig. 1). This lack of effect was not altered in any sensitivity analysis. There was no suggestion of heterogeneity between trials and the funnel plot did not suggest small study bias (Supplementary Fig. 3). Subgrouping did not suggest effect differences by duration, dose, nutrients replaced, intervention type, age, sex or baseline cancer risk. Eighteen trials (99,336 participants) provided data on 2277 cancer deaths (RR 0.97, 95% CI 0.90 to 1.06, I^2^ 0%, Fig. 2). This lack of effect did not alter in sensitivity analyses or subgrouping and there was no suggestion of small study bias (Supplementary Fig. 4) or heterogeneity.

**Fig. 3:**
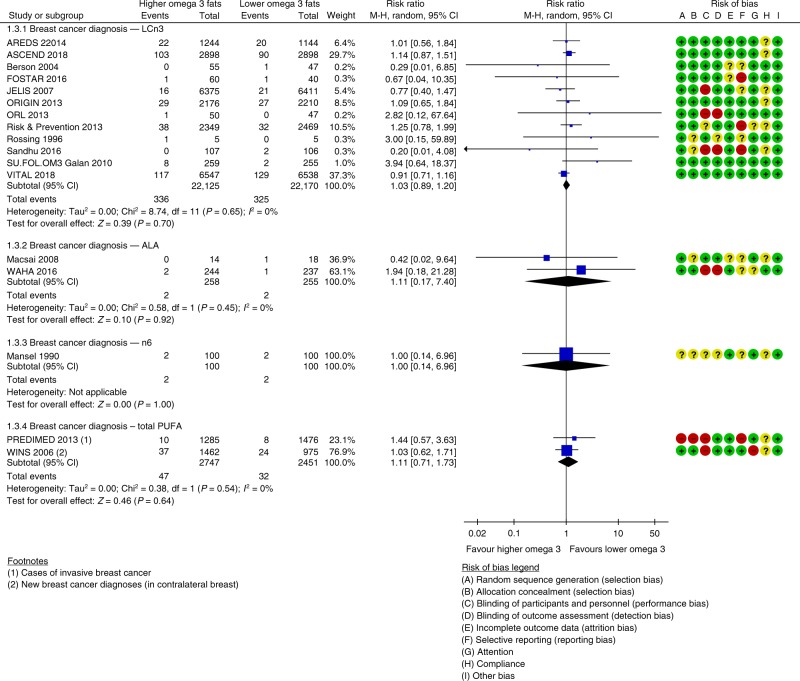
Forest plot showing effects of increasing omega-3, omega-6 and total PUFA on diagnosis of breast cancer in women participants, using random-effects meta-analyses.

Increasing LCn3 probably has little or no effect on risk of breast cancer diagnosis (moderate-quality evidence), but effects on breast cancer deaths are unclear as the evidence is of very low-quality (two deaths). We meta-analysed 12 trials (44,295 women, 661 diagnoses, mean duration 48 months, mean dose 1.9 g/d LCn3) assessing effects of LCn3 on breast cancer diagnosis (RR 1.03, 95% CI 0.89–1.20, I^2^ 0%, Fig. 3). This lack of effect did not alter in sensitivity analyses, there was no suggestion of small study bias or heterogeneity. Subgrouping did not suggest differences in effect by duration, dose, replacement, intervention type, age, sex or cancer risk. Breast density data were consistent with little or no effect.

**Fig. 4:**
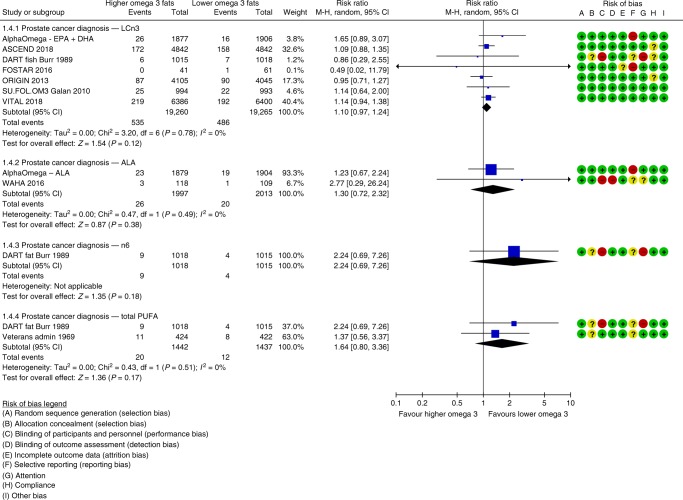
Forest plot showing effects of increasing omega-3, omega-6 and total PUFA on diagnosis of prostate cancer in male participants, using random-effects meta-analyses.

Increasing LCn3 may slightly increase prostate cancer risk (low-quality evidence), but effects on prostate cancer death were unclear (the evidence was very low-quality, five deaths). Seven trials (38,525 men, mean duration 51 months, mean dose 1.2 g/d LCn3) reported on 1021 prostate cancer diagnoses, finding higher risk of prostate cancer in men with increased LCn3 (RR 1.10, 95% CI 0.97–1.24, I^2^ 0%, NNTH 334, Fig. 4). This slight increase in prostate cancer risk was stable to all sensitivity analyses. However, the suggestion of harm was contradicted by PSA data reported in a single large trial (25% reduction, MD −0.13 ng/ml, 95% CI −0.25 to 0.01, 1622 participants). Raised PSA was reported in 12 of 62 participants in another trial (RR 0.47, 95% CI 0.16–1.40), also contradicting the suggested LCn3 harms.

### Effects of increasing ALA

Increasing ALA probably has little or no effect on risk of cancer death (moderate-quality evidence) and may slightly increase the risk of prostate cancer diagnosis (low-quality evidence). Data on any cancer diagnoses, breast cancer diagnoses, breast or prostate cancer deaths and breast density were too limited to provide useful information, so effects were unclear.

Two trials (5545 participants, durations 24 and 40 months, doses 2 and 5 g/d ALA) provided data on 123 cancer deaths and meta-analysis suggested little or no effect (RR 1.05, 95% CI 0.74–1.49, I^2^ 0%), which did not alter in sensitivity analyses. The same two trials reported 46 prostate cancer diagnoses in 4010 male participants (RR 1.30, 95% CI 0.72–2.32, NNTH 334, I^2^ 0%). This increased risk was consistent across all sensitivity analyses and supported by a rise in PSA in those taking more ALA in the single large trial (rise of 23% from baseline, MD 0.10 ng/ml, 95% CI −0.03 to 0.23).

### Effects of increasing omega-6

Evidence for effects of omega-6 on all cancer outcomes was unclear and of very low-quality (see Supplementary Materials).

### Effects of increasing total PUFA

Increasing total PUFA may slightly increase risk of diagnosis of any cancer and cancer death (both low-quality evidence). No trials reported breast cancer deaths or breast density, prostate cancer deaths or PSA and effects on breast and prostate cancer diagnoses were unclear (evidence of very low-quality).

Eight trials (9428 participants, 436 diagnoses, mean duration 39 months, doses ranging from 0.8% of energy to 38% of energy from PUFA) assessed effects of increasing total PUFA on cancer diagnosis (RR 1.19, 95% CI 0.99–1.42, NNTH 125, I^2^ 0%), consistent across sensitivity analysis. While the funnel plot suggested small trials with higher risk ratios may be missing (Supplementary Fig. 10), if such trials were included the risk ratio would increase further. Subgrouping did not suggest important differences due to study duration, PUFA dose, age, sex, baseline cancer risk or replacement but data were limited for assessment of subgroup effects. Four trials (3407 participants, 73 deaths, mean duration 39 months, median dose 7%E from PUFA) reported on cancer deaths (RR 1.10, 95% CI 0.48–2.49, NNTH 500, I^2^ 37%), consistent across all sensitivity analyses.

### Secondary outcomes

Effects on body weight and measures of adiposity are reported as primary outcomes in other reviews in this series.^33–35^ No trials reported on quality of life; dropouts are reported in supplementary materials.

## Discussion

We included 47 long-term RCTs, randomising 108,194 participants. Increasing LCn3 probably has little or no effect on risk of cancer diagnosis, cancer death or breast cancer diagnosis but may slightly increase prostate cancer risk (NNTH 334). Increasing ALA probably has little or no effect on risk of cancer death but may slightly increase prostate cancer risk (NNTH 334). Effects of omega-6 were unclear. Increasing total PUFA may slightly increase risk of diagnosis of any cancer (NNTH 125) and cancer death (NNTH 500).

### Strengths and limitations

Strengths of this systematic review include its large size (47 long-term RCTs including more than 108,000 randomised participants worldwide). Creation of a dataset of RCTs randomising to higher or lower PUFA intakes, regardless of primary and reported outcomes, allowed the inclusion of trials and data that would otherwise have been missed or remained unpublished. This allowed us to include many large and long-term RCTs of PUFAs in populations recruited for health problems other than cancer risk, so allowing us to assess effects of increasing PUFA on diagnosis of cancers in low-risk populations. As meta-analysis of trials with rare events can produce different effect sizes when using different analytical methods we ran sensitivity analyses using Mantel–Haenszel and Peto fixed-effects meta-analyses and compared the results with the main random-effects Mantel–Haenszel analysis.^48–51^ This ensures that review results are robust to analysis methods.

Review limitations include limited available data on effects of increasing ALA, omega-6 and total PUFA. It was notable that doses of total PUFA were highly variable (from 0.8% of energy to almost 38% of energy from total PUFA in trials providing cancer diagnosis data), but the small number of trials made subgrouping by dose uninformative (Supplementary Figure 11). LCn3 results resulted from meta-analyses of mainly supplementary trials, so effects of increasing oily fish consumption are unclear. As poorly concealed allocation is associated with a 40% greater effect size^52^ and lack of blinding with additional bias^53,54^ caution is needed in interpreting small effects in weaker trials. As prostate cancer was not a primary outcome in this review, we did not ask trialists for additional prostate cancer data, which means that more information on prostate cancer may be available from existing trials.

### What does this study add?

Our review concurs with a systematic review of observational data^23^ and two including fewer trials (10 and 19 RCTs to our 34) suggesting LCn3 intake is not associated with total cancer risk.^15,16^ Two previous systematic reviews of trials and observational data suggested there were inadequate data to determine whether LCn3 intake was associated with prostate cancer incidence or progression.^26,29^ A systematic review of cohort studies assessing relationships between omega-3 and eleven types of cancer found mixed results, including cohorts suggesting both statistically significantly increased and decreased risk for prostate cancer.^23^ This review is new in suggesting that actively increasing dietary total PUFA may slightly increase the risk of both cancer diagnosis and cancer mortality. A recent systematic review of observational studies suggested no association between total polyunsaturated fat intake and breast cancer risk,^22^ but as higher PUFA intake is associated with healthier lifestyles small harms may be difficult to spot in observational studies due to confounding. RCT data are insufficient to corroborate or contradict two nested case-control trials suggesting that higher PUFA intake correlates to higher prostate cancer risk.^24,25^

**Fig. 5:**
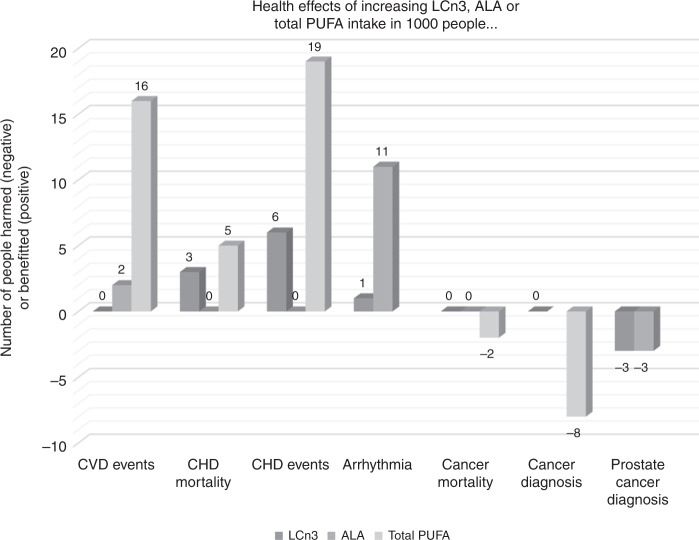
Visual representation of number of additional diagnoses or deaths incurred or avoided per 1000 people increasing their LCn3, ALA or total PUFA intake across cancer and cardiovascular outcomes. Bars above zero suggest the number of people who would benefit of 1000 people consuming more PUFA (LCn3, ALA or total PUFA), bars below zero suggest the number of people who would be harmed of 1000 people consuming more PUFA. Where the evidence suggests little or no effect zero appears, and where the evidence is of very low quality no data appear. Cancer data are from this review, CVD data from sister Cochrane reviews.^34,55^

The small harms resulting from increased LCn3, ALA and total PUFAs need to be balanced against potential gains from the other major cause of morbidity and mortality, cardiovascular disease (Table 1). For example, this review suggests that increasing LCn3 intake may increase the risk of prostate cancer in men, such that 1000 men increasing their LCn3 intake would lead to three additional men being diagnosed with prostate cancer. In a sister review, meta-analysis including 25 RCTs and over 127,000 participants suggests that if 1000 people consume more LCn3 three will avoid death from coronary heart disease. Further analyses suggest that of the 1000 six will avoid a CHD event and one will avoid arrhythmia.^55^ The balance appears similar for ALA—for every 1000 people increasing their ALA intake two will avoid a CVD event, eleven will avoid arrhythmia but three will be diagnosed with prostate cancer who would not otherwise have been diagnosed (Fig. 5 represents the harms and benefits visually as number of additional diagnoses incurred or avoided per 1000 people increasing their LCn3, ALA or total PUFA intake).^55^ Increasing total PUFA in 1000 people appears to prevent five people dying from CHD, but two additional people will die from cancer. Sixteen people will be protected from CVD events, nineteen from CHD events, but eight more will be diagnosed with cancer (Fig. 5).^34^ This suggests that small benefits and small harms of increasing LCn3 intake are likely to be partially balanced out across major sources of morbidity and mortality and indeed increasing LCn3, ALA, omega-6 and total PUFA appear to have little or no effect on all-cause mortality (Table 1).^34,35,55^

**Table 1:** Table comparing effects of LCn3, ALA, omega-6 and total PUFA on key cardiovascular outcomes and cancer outcomes from reviews within this WHO series.

Key outcomes	Effects of increased…. [RR (95% CI), number of participants, number of RCTs, GRADE level of evidence & summary]			
	Long-chain omega-3	Alpha-linolenic acid	Omega-6	Total PUFA
Mortality	RR 0.97 (0.93–1.01) 143,693 participants, 45 RCTs GRADE: High quality evidence of little or no effect^55^	RR 1.01 (0.84–1.20) 19,327 participants, 5 RCTs GRADE: Moderate quality evidence of little or no effect^55^	RR 1.00 (0.88–1.12) 4506 participants, 10 RCTs GRADE: Low quality evidence of little or no effect^35^	RR 0.98 (0.89–1.07) 19,290 participants, 24 RCTs GRADE: Moderate quality evidence of little or no effect^34^
CVD: CVD mortality	RR 0.92 (0.86–0.99) 117,837 participants, 29 RCTs GRADE: Moderate quality evidence of little or no effect^55^	RR 0.96 (0.74–1.25) 18,619 participants, 4 RCTs GRADE: Moderate quality evidence of little or no effect^55^	RR 1.09 (0.76–1.55) 4019 participants, 7 RCTs GRADE: Very low quality, effect of omega-6 on CVD mortality is unclear^35^	RR 1.02 (0.82–1.26) 15,107 participants, 16 RCTs GRADE: Low quality evidence of little or no effect^34^
CVD: CVD events	RR 0.96 (0.92–1.01) 140,482 participants, 43 RCTs GRADE: High quality evidence of little or no effect^55^	RR 0.95 (0.83–1.07) 19,327 participants, 5 RCTs GRADE: Low quality evidence that increasing ALA may reduce CVD event risk (NNTB 500, 95% CI NNTB 125 to NNTH 334)^55^	RR 0.97 (0.81–1.15) 4962 participants, 7 RCTs GRADE: Low quality evidence of little or no effect^35^	RR 0.89 (0.79–1.01) 17,799 participants, 21 RCTs GRADE: Moderate quality evidence that increasing PUFA reduces CVD events (NNTB 63, 95% CI NNTB 33 to NNTH 1000)^34^
CVD: CHD mortality	RR 0.90 (0.80–1.00) 127,667 participants, 25 RCTs GRADE: Low quality evidence that increasing LCn3 reduces CHD mortality (NNTB 334, 95% CI NNTB 200 to NNTB ∞ )^55^	RR 0.95 (0.72–1.26) 18,353 participants, 3 RCTs GRADE: Moderated quality evidence of little or no effect^55^	Not assessed^35^	RR 0.91 (0.78–1.06) 8810 participants, 9 RCTs GRADE: Low quality evidence that increasing PUFA reduces CHD mortality (NNTB 200, 95% CI NNTB 72 to NNTH 250)^34^
CVD: CHD events	RR 0.91 (0.85–0.97) 134,405 participants, 33 RCTs GRADE: Low quality evidence that increasing LCn3 may reduce risk of CHD events (NNTB 167, 95% CI NNTB 100 to NNTB 500)^55^	RR 1.00 (0.82–1.22) 19,061 participants, 4 RCTs GRADE: Low quality evidence of little or no effect^55^	RR 0.88 (0.66–1.17) 3997 participants, 7 RCTs GRADE: Very low, effect of omega-6 on CHD events is unclear^35^	RR 0.87 (0.72–1.06) 10,076 participants, 15 RCTs GRADE: Moderate quality evidence that increasing PUFA reduces risk of CHD events (NNTB 53, 95% CI NNTB 25 to NNTH 167)^34^
CVD: stroke	RR 1.02 (0.94–1.12) 138,888 participants, 31 RCTs GRADE: Moderate quality evidence of little or no effect^55^	RR 1.15 (0.66–2.01) 19,327 participants, 5 RCTs GRADE: Very low, effect of ALA on stroke is unclear^55^	RR 1.36 (0.45–4.11) 3730 participants, 4 RCTs GRADE: Very low, effect of omega-6 on stroke is unclear^35^	RR 0.91 (0.58–1.44) 14,742 participants, 11 RCTs GRADE: Low quality evidence that increasing PUFA reduces stroke risk slightly (NNTB 1000, 95% CI NNTB 200 to NNTH 167)^34^
Cancer: any cancer diagnosis	RR 1.02 (0.98–1.07) 113,557 participants, 27 RCTs GRADE: High quality evidence of little or no effect	RR 0.98 (0.38–2.55) 752 participants, 2 RCTs GRADE: Very low, effect of ALA on cancer diagnosis is unclear	RR 1.21 (0.96–1.53) 4272 participants, 6 RCTs GRADE: Very low, effect of omega-6 on cancer diagnosis is unclear	RR 1.19 (0.99–1.42) 9428 participants, 8 RCTs GRADE: Low quality evidence that increasing total PUFA may increase risk of cancer diagnosis (NNTH 125, 95% CI NNTB ∞ to NNTH 59)
Cancer: breast cancer diagnoses	RR 1.03 (0.89–1.20) 44,295 participants, 12 RCTs GRADE: Moderate quality evidence of little or no effect	RR 1.11 (0.17– 7.40) 513 participants, 2 RCTs GRADE: Very low, effect of ALA on breast cancer diagnosis is unclear	RR 1.00 (0.14–6.96) 200 participants, 1 RCT GRADE: Very low, effect of omega-6 on breast cancer diagnosis is unclear	RR 1.11 (0.71–1.73) 5198 participants, 2 RCTs GRADE: Very low, effect of total PUFA on breast cancer diagnosis is unclear
Cancer: prostate cancer diagnoses	RR 1.10 (0.97–1.24) 38,525 participants, 7 RCTs GRADE: Low quality evidence that increasing LCn3 may increase prostate cancer risk (NNTH 334, 95% CI NNTB 1000 to NNTH 167)	RR 1.30 (0.72– 2.32) 4010 participants, 2 RCTs GRADE: Low quality evidence that increasing ALA may increase prostate cancer risk (NNTH 334, 95% CI NNTB 334 to NNTH 77)	RR 2.24 (0.69–7.26) 2033 participants, 1 RCT GRADE: Very low, effect of omega-6 on prostate cancer diagnosis is unclear	RR 1.64 (0.80–3.36) 2879 participants, 2 RCTs GRADE: Very low, effect of total PUFA on prostate cancer diagnosis is unclear

NNTB: the number of people needed to increase their PUFA intake for one additional person to benefit.

NNTH: the number of people needed to be increase their PUFA intake for one additional person to be harmed.

While increasing LCn3 has little or no effect on risk of cancer diagnosis, breast cancer diagnosis or cancer death (moderate and high-quality evidence), trial evidence suggests that increasing omega-3 may slightly increase prostate cancer risk, and increasing total PUFA may slightly increase cancer risk (low-quality evidence), although this could result from very high intakes of PUFA in some trials. Considering both cancer and cardiovascular outcomes, overall health effects of increasing LCn3, ALA, omega-6 and total PUFA appear small.

## Supplementary information

Supplementary file Ca & PUFA

## Data Availability

The dataset for this review was part of our published dataset, and so is publicly available, see ref. ^39^
