# Supplementary material for: Omega-3, omega-6 and total dietary polyunsaturated fat on cancer incidence: systematic review and meta-analysis of randomised trials
Source: Br J Cancer. 2020 Feb 29;122(8):1260–70. doi: 10.1038/s41416-020-0761-6 (PMC7156752; doi:10.1038/s41416-020-0761-6)
Supplement: Supplementary file 1 — Supplementary file Ca & PUFA [file 41416_2020_761_MOESM1_ESM.docx]

**Supplementary file for Hanson et al:**

**Effects of supplementary dietary polyunsaturated fat on cancer incidence: systematic review and meta-analysis of randomised trials**

Contents

[RESULTS 3](#_Toc25835715)

[Effects of long-chain omega-3 fat on risk of any cancer 3](#_Toc25835716)

[Effects of long-chain omega-3 fat on risk of breast cancer 4](#_Toc25835717)

[Effects of long-chain omega-3 fat on prostate cancer 5](#_Toc25835718)

[Effects of ALA on risk of any cancer 5](#_Toc25835719)

[Effects of ALA on risk of breast cancer 6](#_Toc25835720)

[Effects of ALA on risk of prostate cancer 6](#_Toc25835721)

[Effects of omega-6 on risk of any cancer 6](#_Toc25835722)

[Effects of omega-6 on risk of breast and prostate cancer 7](#_Toc25835723)

[Effects of total PUFA on risk of any cancer 7](#_Toc25835724)

[Effects of total PUFA on risk of breast cancer 8](#_Toc25835725)

[Effects of total PUFA on risk of prostate cancer 9](#_Toc25835726)

[Secondary outcomes 9](#_Toc25835727)

[Supplementary Figure 1: PRISMA flow diagram for this review 10](#_Toc25835728)

[Supplementary Figure 2: Summary risk of bias of included comparisons by domain as assessed by reviewers. 11](#_Toc25835729)

[Supplementary Figure 3. Funnel plot for effects of LCn3 on diagnosis of any cancer. 12](#_Toc25835730)

[Supplementary Figure 4. Funnel plot for effects of LCn3 on death from any cancer. 12](#_Toc25835731)

[Supplementary Figure 5. Funnel plot for effects of LCn3 on diagnosis of breast cancer. 13](#_Toc25835732)

[Supplementary Figure 6. Forest plot showing effects of increasing omega-3, omega-6 and total PUFA on deaths from breast cancer in women participants, using random-effects meta-analyses. 14](#_Toc25835733)

[Supplementary Figure 7. Forest plot showing effects of increasing omega-3, omega-6 and total PUFA on breast density in cm^2^, using random-effects meta-analyses. 14](#_Toc25835734)

[Supplementary Figure 8. Forest plot showing effects of increasing omega-3, omega-6 and total PUFA on deaths from prostate cancer in male participants, using random-effects meta-analyses. 15](#_Toc25835735)

[Supplementary Figure 9. Forest plot showing effects of increasing omega-3, omega-6 and total PUFA on prostate specific antigen (PSA, ng/ml), using random-effects meta-analyses. 15](#_Toc25835736)

[Supplementary Figure 10. Funnel plot for effects of total PUFA on diagnosis of any cancer. 16](#_Toc25835737)

[Supplementary Figure 11. Meta-analysis assessing effects of increasing total PUFA on diagnosis of any cancer, subgrouping by dose of PUFA (as percentage of energy intake). 17](#_Toc25835738)

[Supplementary Figure 12. Forest plot showing effects of increasing LCn3 on side effects using random-effects meta-analyses. 18](#_Toc25835739)

[Supplementary Figure 13. Forest plot showing effects of increasing LCn3 on dropouts using random-effects meta-analyses. 19](#_Toc25835740)

[Supplementary Figure 14. Forest plot showing effects of increasing ALA on side effects using random-effects meta-analyses 19](#_Toc25835741)

[Supplementary Figure 15. Forest plot showing effects of increasing omega-6 on dropouts using random-effects meta-analyses. 20](#_Toc25835742)

[Supplementary Table 1. Table of characteristics, risk of bias and references for included trials 21](#_Toc25835743)

[Supplementary Table 2. High vs low LCn3 (primary outcomes) 26](#_Toc25835744)

[Supplementary Table 3. High vs low LCn3 (secondary outcomes) 31](#_Toc25835745)

[Supplementary Table 4. GRADE table: summary of findings of effects of omega-3 fats (LCn3 and ALA) on cancers 32](#_Toc25835746)

[Supplementary Table 5. High vs low ALA (primary outcomes) 34](#_Toc25835747)

[Supplementary Table 6. High vs low ALA (secondary outcomes) 36](#_Toc25835748)

[Supplementary Table 7. High vs low omega-6 (primary outcomes) 37](#_Toc25835749)

[Supplementary Table 8. High vs low omega-6 (secondary outcomes) 39](#_Toc25835750)

[Supplementary Table 9. GRADE table: summary of findings of effects of omega-6 fats on cancers 40](#_Toc25835751)

[Supplementary Table 10. High vs low total PUFA (primary outcomes) 41](#_Toc25835752)

[Supplementary Table 11. High vs low total PUFA (secondary outcomes) 44](#_Toc25835753)

[Supplementary Table 12. GRADE table: summary of findings of effects of total PUFA on cancers 45](#_Toc25835754)

[References 46](#_Toc25835755)

# RESULTS

**(in greater detail than the main paper)**

From our trials database we included 47 RCTs (49 comparisons, including 108,194 participants) that assessed outcomes of interest to this review. Thirty four trials (including 97,548 participants) assessed effects of LCn3, three (3179 participants) assessed effects of ALA, eight (4976 participants) assessed effects of omega-6 and 9 trials (including 11,573 participants) assessed effects of total PUFA (Supplementary Figure 1, Supplementary Table 1). Several trials assessed more than one of these interventions, so numbers of trials and participants are not additive. Of the 47 trials, 38 included participants with normal baseline cancer risk (including healthy adults and those with risk factors for other diseases, or existing disease including CVD, diabetes and eye diseases), 3 included participants with cancer risk factors (2 at high risk of breast cancer, 1 at high risk of bowel cancer) and 6 included participants with previously diagnosed cancer (1 postoperative breast cancer, 3 postoperative colorectal cancer, 1 prostate cancer, one skin cancer). Most trials provided supplementary capsules, but trials of omega-6 and total PUFA tended to provide dietary advice with or without supplementary foods, some trials provide supplementary foods (such as enriched margarines, nuts, and one (set in an institution) provided all food. In four trials the intervention was to reduce fat intake, which also reduced PUFA, so for these trials the higher PUFA arm was the study control arm. Mean trial duration was over 30 months, and most trials were conducted in Europe (20 trials) or North America (15 trials), five were conducted in Japan, two in Australia and/or New Zealand, and five were conducted over more than one continent. Seventeen of the 47 trials were at low summary risk of bias (Supplementary Figure 2, Supplementary Table 1).

## Effects of long-chain omega-3 fat on risk of any cancer

Effects of LCn3 on all primary and secondary outcomes, along with sensitivity analyses and subgroupings are displayed in Supplementary Tables 2 and 3. The GRADE assessment is shown in Supplementary Table 4.

Meta-analysis of 27 trials (113,557 participants) reporting from 1 to 1784 cancer diagnoses suggested little or no effect on any cancer diagnosis (RR 1.02, 95% CI 0.98 to 1.07, I^2^ 0%, Figure 1 in the main paper, high quality evidence), and this lack of effect did not alter in fixed effects meta-analysis, when limiting to trials at low summary risk of bias, low risk of compliance issues or larger trials (at least 100 randomised participants). There was no suggestion of heterogeneity between trials and the funnel plot did not suggest small study bias (Supplementary Figure 3). Subgrouping did not suggest differences in effect by duration, dose, nutrients replaced by LCn3, intervention type, age, sex or baseline cancer risk (test for subgroup differences all p>0.05). Mean duration of included trials was 32 months (SD 22, range 12 to 88 months) and mean dose of LCn3 was 1.7g/d (SD 1.2g/d, range 0.5 to 4.6g/d). Increasing LCn3 has little or no effect on risk of diagnosis of any cancer (high quality evidence).

Eighteen trials (99,336 participants) provided data on 2277 cancer deaths and meta-analysis suggested little or no effect of increasing LCn3 (RR 0.97, 95% CI 0.90 to 1.06, I^2^ 0%, Figure 2 in the main paper), and the lack of effect didn’t alter in any sensitivity analysis. Subgrouping did not suggest differential effects by trial duration, LCn3 dose, replacement for LCn3, intervention type, age, sex or baseline cancer risk. There was no suggestion of heterogeneity between trials, and the funnel plot showed no sign of small study bias (Supplementary Figure 4). Mean duration of included trials was 43 months (SD 22, range 12 to 88 months) and mean dose of LCn3 was 1.6g/d (SD 1.5g/d, range 0.4 to 6.0g/d). Increasing LCn3 probably has little or no effect on risk of cancer death (moderate quality evidence, downgraded once for imprecision).

## Effects of long-chain omega-3 fat on risk of breast cancer

Meta-analysis of 12 trials (92,736 participants, 44,304 women) reporting from 1 to 246 breast cancer diagnoses (661 diagnoses overall) suggest little or no effect of LCn3 on breast cancer diagnosis (RR 1.03, 95% CI 0.89 to 1.20, I^2^ 0%, Figure 3 in the main paper), and this lack of effect did not alter in fixed effects meta-analysis, when limiting to trials at low summary risk of bias, low risk of compliance issues or larger trials (at least 100 randomised participants). There was no suggestion of heterogeneity between trials. Subgrouping did not suggest differences in effect by duration, dose, nutrients replaced by LCn3, intervention type, age, sex or baseline cancer risk (test for subgroup differences all p>0.05), however trials tended to cluster into specific subgroups rather than be spread evenly across subgroups, so differences would be harder to see. There was no suggestion of small study bias in the funnel plot (Supplementary Figure 5). Mean duration of included trials was 48 months (SD 25, range 12 to 88 months) and mean dose of LCn3 was 1.9g/d (SD 1.5g/d, range 0.6 to 4.6g/d). Increasing LCn3 probably has little or no effect on risk of breast cancer diagnosis (moderate quality evidence, downgraded once for imprecision).

Two trials (including 3322 participants, 102 women) reported breast cancer deaths, but each reported a single death, so there were insufficient data to assess effects (Supplementary Figure 6). One of the included trials is a male only study but we included the data in Supplementary Figure 6 as it reported a single death from breast cancer ^1^, men are not included in other breast cancer trials. Mean duration of included trials was 60 months (SD 17, range 48 to 72 months) and mean dose of LCn3 was 0.9g/d (SD 0.5g/d, range 0.5 to 1.2g/d). The effect of increasing LCn3 on breast cancer deaths is unclear as the evidence is of very low quality (downgraded once for risk of bias, twice for imprecision).

Lower breast density is associated with lower risk of breast cancer in women. A single trial (not at low summary risk of bias) of 175 women reported on breast density, suggesting a mean difference of 2.06cm^2^ (95% CI -4.68 to 8.81, Supplementary Figure 7), a change of less than 10% from the control group baseline of 56cm^2^. This did not change in fixed effects sensitivity analysis or retaining trials of at least 100 participants, but the single trial was lost in sensitivity analyses on summary risk of bias and risk from compliance problems. The effect of increasing LCn3 was unclear as the evidence was of very low quality (downgraded once for imprecision and risk of bias, downgraded twice for indirectness).

## Effects of long-chain omega-3 fat on prostate cancer

Seven trials (63,460 participants, 38,525 men) reported on 1021 prostate cancer diagnoses, finding higher risk of prostate cancer in men with increased LCn3 (RR 1.10, 95% CI 0.97 to 1.24, I^2^ 0%, Figure 4 in the main paper). This slight increase in prostate cancer risk was stable to all sensitivity analyses. With so few trials we did not carry out subgrouping or assess funnel plots. However, the suggestion of harm was contradicted by findings on PSA (below). Mean duration of included trials was 51 months (SD 24, range 24 to 88 months) and mean dose of LCn3 was 1.2g/d (SD 1.5g/d, range 0.4 to 4.5g/d). Increasing LCn3 may increase the risk of prostate cancer (low quality evidence, downgraded once each for imprecision and inconsistency).

Prostate cancer deaths were reported in only two trials (5 deaths in 5616 participants, 5101 men, Supplementary Figure 8) so effects of LCn3 on prostate cancer deaths could not be assessed. The trials were of 48 and 72 months duration, doses of LCn3 were 0.5 and 0.6g/d. The effect of increasing LCn3 on prostate cancer death is unclear as the evidence is of very low quality (downgraded once for inconsistency and twice for imprecision).

Prostate specific antigen (PSA) is a marker of prostate cancer risk, and higher PSA is associated with higher risk. PSA was reported as a continuous measure in a single large trial of 1622 participants (at low summary risk of bias, MD -0.13ng/ml, 95% CI -0.25 to 0.01, Supplementary Figure 9), suggesting a fall of 25% from a baseline of 0.53ng/ml in those on higher LCn3. Odds of increased PSA was reported in a single trial (not at low summary risk of bias) of 62 participants, reporting only 12 participants with raised PSA (RR 0.47, 95% CI 0.16 to 1.40, Supplementary Table 2), but also suggesting protective effects of LCn3 on PSA.

## Effects of ALA on risk of any cancer

Effects of ALA on all primary and secondary outcomes, along with sensitivity analyses and subgroupings are displayed in Supplementary Tables 5 and 6. The GRADE assessment is shown in Supplementary Table 4.

Meta-analysis of 2 trials (752 participants) reported 16 cancer diagnoses and suggested little or no effect on risk of cancer diagnosis (RR 0.98, 95% CI 0.38 to 2.55, I^2^ 0%, Figure 1 in the main paper), and this lack of effect did not alter in fixed effects meta-analysis, but no trials were at low summary risk of bias. The single large trial (with >100 participants) was also the single trial at low risk of compliance issues and suggested a slight increase in cancer risk with increased ALA (RR 1.09, 95% CI 0.40 to 2.98) but with very wide confidence intervals. As there were only two trials we did not attempt subgrouping or a funnel plot. Mean duration of included trials was 18 months (12 and 24 months), mean dose of LCn3 was 4.2g/d (3.3 and 5.0g/d). The effect of increasing ALA on diagnosis of any cancer is unclear as the evidence was of very low quality (downgraded once for risk of bias, twice for imprecision).

Two trials (5545 participants) provided data on 123 cancer deaths and meta-analysis suggested little or no effect of LCn3 (RR 1.05, 95% CI 0.74 to 1.49, I^2^ 0%, Figure 2 in the main paper), which didn’t alter in any sensitivity analysis. Subgrouping and funnel plots re not attempted. Duration of included trials was 24 and 40 months, doses of LCn3 were 2 and 5g/d. Increasing ALA probably has little or no effect on risk of cancer death (moderate quality evidence, downgraded once for imprecision).

## Effects of ALA on risk of breast cancer

Two trials (752 participants, 513 women) reported only 4 breast cancer diagnoses, and no trials reported deaths from breast cancer or breast density, so there were insufficient data to assess effects on breast cancer diagnoses, deaths or markers (Figure 3 in the main paper and Supplementary Figure 6). Duration of included trials of breast cancer diagnosis was 12 and 24 months, doses of LCn3 were 3.3 and 5.0g/d. The effect of increasing ALA on risk of breast cancer diagnosis is unclear as the evidence is of very low quality (downgraded once for risk of bias, twice for imprecision),

## Effects of ALA on risk of prostate cancer

Meta-analysis of 2 trials (5545 participants, 4010 men) reporting 46 prostate cancer diagnoses suggesting that increasing ALA increases risk of prostate cancer diagnosis (RR 1.30, 95% CI 0.72 to 2.32, I^2^ 0%, Figure 4 in the main paper). This increase in risk was consistent across all sensitivity analyses, and supported by a rise in PSA with ALA (below). Mean duration of included trials was 32 months (24 and 40 months), mean dose of LCn3 was 3.5g/d (2.0 and 5.0g/d). Increasing ALA may increase the risk of prostate cancer diagnosis (low quality evidence, downgraded twice for imprecision).

No trials reported deaths from prostate cancer (Supplementary Figure 8). A single large trial at low summary risk of bias reported increased risk of raised PSA (>4ng/ml, RR 1.13, 95% CI 0.86 to 1.50) and higher PSA (by 23% from baseline, MD 0.10ng/ml, 95% CI -0.03 to 0.23, Supplementary Figure 9) in those taking more ALA.

## Effects of omega-6 on risk of any cancer

Effects of omega-6 on all primary and secondary outcomes, along with sensitivity analyses and subgroupings are displayed in Supplementary Tables 7 and 8. The GRADE assessment is shown in Supplementary Table 9.

Six trials (4272 participants, 262 cancer diagnoses) suggested that increasing omega-6 increased risk of diagnosis of any cancer (RR 1.21, 95% CI 0.96 to 1.53, I^2^ 0%, Figure 1 in the main paper). The increased risk was consistent between dietary and supplemental interventions, and in all sensitivity analyses except when restricting to the single trial at low summary risk of bias. Mean duration of the included trials was 30 months (SD 25, range 12 to 72 months), mean dose was 10.7%E from omega-6, but varied enormously (SD 13.9, median 6.4%E, range 0.2 to 37.8%E from omega-6). The effect of increasing omega-6 on cancer diagnosis is unclear as the evidence is of very low quality (downgraded twice for risk of bias, once for imprecision).

Meta-analysis of the four trials assessing effects of omega-6 on cancer deaths was heterogeneous, and suggested little or no effect (RR 0.97, 95% CI 0.51 to 1.85, I^2^ 52%, Figure 2 in the main paper). However, none of the trials were at low summary risk of bias, and fixed effects analysis suggested in increase in risk of cancer death. No subgrouping or funnel plots were run as we included few trials. Mean duration of the included trials was 37 months (SD 12, range 24 to 48 months), mean dose was 14.0%E from omega-6, but varied a great deal (SD 21.0, median 2.8%E, range 1.4 to 37.8%E from omega-6). The effect of omega-6 on cancer deaths is unclear as the evidence is of very low quality (downgraded once each for risk of bias, imprecision and inconsistency).

## Effects of omega-6 on risk of breast and prostate cancer

Only one small trial (200 women participants, 4 breast cancer diagnoses, 12 months duration, 2.7%E from omega-6, Figure 3) assessed effects of omega-6 on breast cancer diagnosis, and none on breast cancer deaths or breast density (Supplementary Figures 6 and 7), so there were insufficient data to assess effects. The effect of omega-6 on breast cancer diagnoses is unclear as the evidence is of very low quality (downgraded once for risk of bias, once for indirectness and twice for imprecision).

One trial (2033 male participants, 24 months duration, 2.8% E increase in omega-6) that was not at low summary risk of bias reported 13 prostate cancer diagnoses (RR 2.24, 95% CI 0.69 to 7.26, Figure 4 in the main paper), no trials reported prostate cancer deaths or PSA. The effect of omega-6 on risk of prostate cancer diagnosis is unclear as the evidence is of very low quality (downgraded once each for risk of bias, indirectness and imprecision).

## Effects of total PUFA on risk of any cancer

Effects of total PUFA on all primary and secondary outcomes, along with sensitivity analyses and subgroupings are displayed in Supplementary Tables 10 and 11. The GRADE assessment is shown in Supplementary Table 12.

Eight trials (9428 participants, 436 diagnoses) assessed effects of increasing total PUFA on cancer diagnosis, suggesting that increasing total PUFA increases diagnosis risk (RR 1.19, 95% CI 0.99 to 1.42, I^2^ 0%, Figure 1 in the main paper). This was consistent across all sensitivity analysis (except when limiting to the three trials at low summary risk of bias, where the RR was 1.08). The funnel plot is difficult to assess with only 8 included trials, but does suggest that smaller trials with higher RRs may be missing (Supplementary Figure 10). If such trials were added back in the RR would rise further. Subgrouping did not suggest important differences between subgroups by study duration, PUFA dose (Supplementary Figure 11), replacement, age, sex and baseline cancer risk. Mean duration of the included trials was 39 months (SD 24, range 12 to 72 months), mean dose was 9.6%E from total PUFA, median 3.3%E, and varied considerably (SD 13, range 0.8 to almost 38%E from total PUFA). Increasing total PUFA may increase risk of diagnosis of any cancer (downgraded once each for risk of bias and imprecision).

Four trials reported on cancer deaths (3407 participants, 73 deaths), suggesting that increasing total PUFA increases risk of death from cancer (RR 1.10, 95% CI 0.48 to 2.49, I^2^ 37%, Figure 2 in the main paper). This increase in risk of cancer death was consistent across all sensitivity analyses. We did not carry out subgrouping or funnel plots as there were only four trials. Mean duration of the included trials was 39 months (SD 27, range 12 to 72 months), mean dose was 13%E from total PUFA, median 7%E, and varied considerably (SD 17, range 0.8 to almost 38%E from total PUFA). Increasing total PUFA may increase the risk of cancer death (downgraded twice for imprecision).

## Effects of total PUFA on risk of breast cancer

Meta-analysis of two trials (5198 female participants, 79 diagnoses) suggested that increasing total PUFA increases risk of breast cancer diagnosis, but with very wide confidence intervals (RR 1.11, 95% CI 0.71 to 1.73, I^2^ 0%, Figure 3 in the main paper). However, this was not supported in sensitivity analysis limiting to the single trial at low summary risk of bias, and neither trial was at low risk of compliance problems. Duration of both trials was 60 months and the dose was 2%E from total PUFA in one trial, unclear in the other. The effect of increasing total PUFA on risk of breast cancer diagnosis is unclear as the evidence is of very low quality (downgraded once for risk of bias and twice for imprecision).

No trials reported breast cancer deaths or breast density (Supplementary Figures 6 & 7).

## Effects of total PUFA on risk of prostate cancer

Meta-analysis of two trials (2879 male participants, 32 diagnoses) suggested that increasing total PUFA increases risk of prostate cancer diagnosis, but with the small number of diagnoses, confidence intervals were very wide (RR 1.64, 95% CI 0.80 to 3.36, I^2^ 0%, Figure 4 in the main paper). No trials were at low summary risk of bias, all other sensitivity analyses suggested increased prostate cancer risk with increased total PUFA. Duration of the included trials was 24 and 72 months, doses 3 and 11%E from total PUFA. The effect of increasing total PUFA on prostate cancer diagnosis is unclear as the evidence is of very low quality (downgraded once for risk of bias and twice for imprecision).

No trials reported prostate cancer deaths or PSA (Supplementary Figures 8 & 9).

## Secondary outcomes

Prostate cancer diagnoses and deaths are reported above. Effects on body weight and measures of adiposity are reported in full (not just in this subset of trials assessing cancer outcomes) in other reviews in this series so are noted in the Supplementary Tables, but not discussed further here.^2-4^ We found no trials reporting any measure of quality of life as effects of increases in LCn3, ALA, omega-6 or total PUFA.

When increasing LCn3 risks of gastrointestinal side effects (RR 1.11, 95% CI 0.89 to 1.31, I^2^ 84%, including effects on nausea, reflux, diarrhoea and hospitalisation for gastrointestinal problems), bleeding (RR 1.09, 95% CI 0.70 to 1.70, I^2^ 59%), and dropouts due to side effects (RR 1.31, 95% CI 0.98 to 1.76, I^2^ 19%) appear increased, while risk of headache or migraine (RR 0.81, 95% CI 0.48 to 1.36, I^2^ 0%), and psychiatric problems (RR 0.70, 95% CI 0.32 to 1.54, I^2^ 0%), appear reduced (Supplementary Figure 12). Overall giving LCn3 appears to have little or no effect on risk of all side effects combined (RR 1.03, 95% CI 0.93 to 1.15, I^2^ 85%), or dropouts for any reason (RR 0.98, 95% CI 0.88 to 1.10, I^2^ 33%, Supplementary Figures 12 and 13).

Data on side effects and dropouts are much more limited for ALA (Supplementary Figure 14) and omega-6 (Supplementary Figure 15, all data on side effects and dropouts shown), and no data were available for trials of total PUFA.


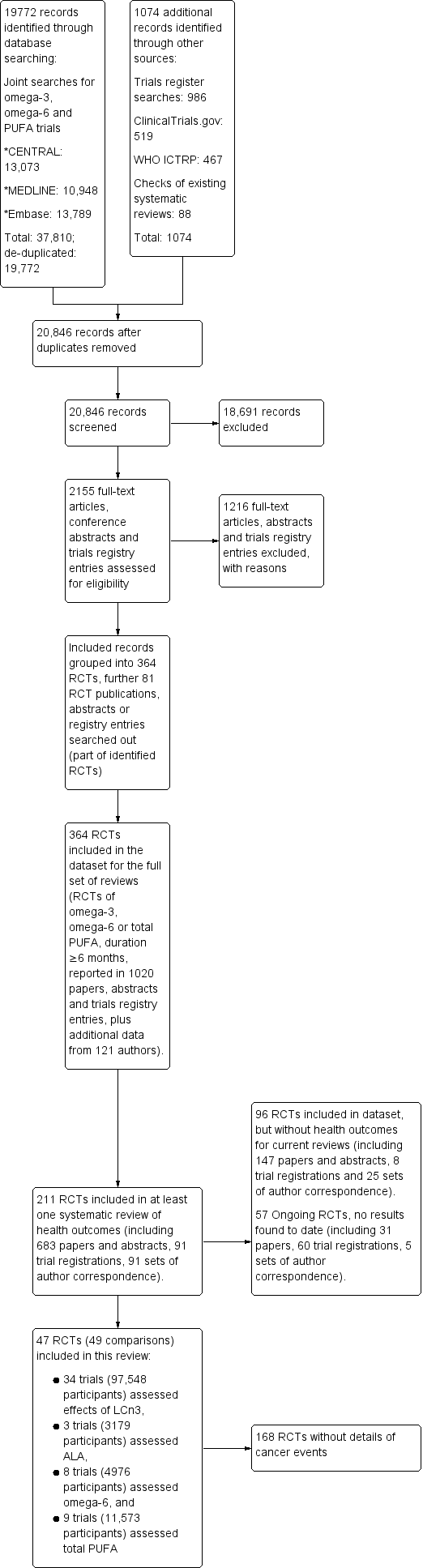


### Supplementary Figure 1: PRISMA flow diagram for this review


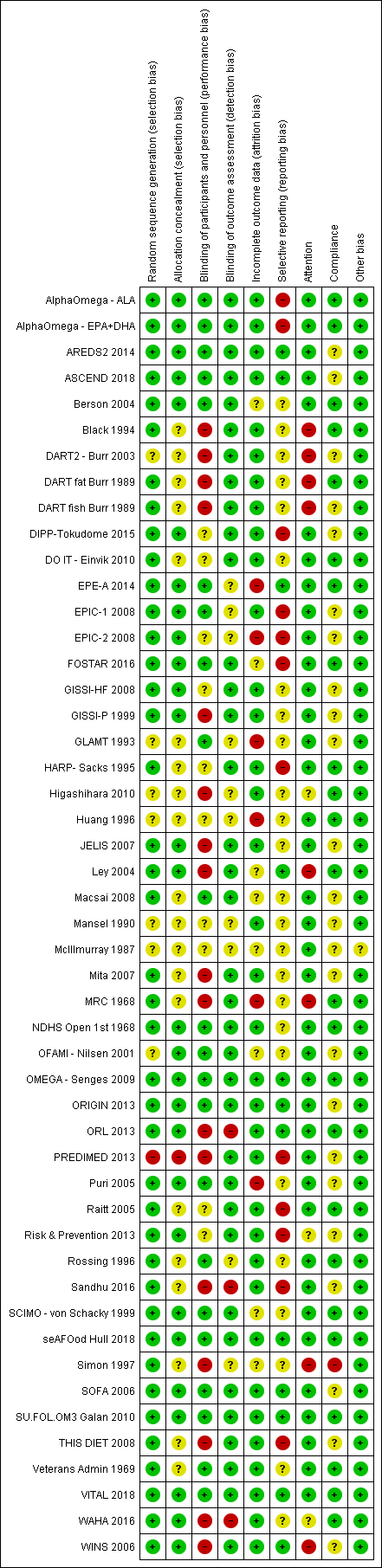


### Supplementary Figure 2: Summary risk of bias of included comparisons by domain as assessed by reviewers.


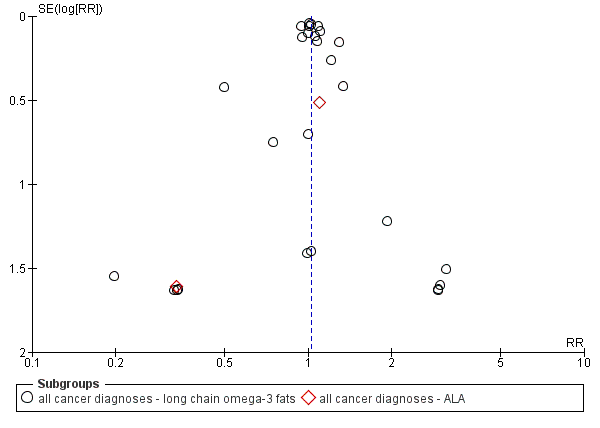


### Supplementary Figure 3. Funnel plot for effects of LCn3 on diagnosis of any cancer.


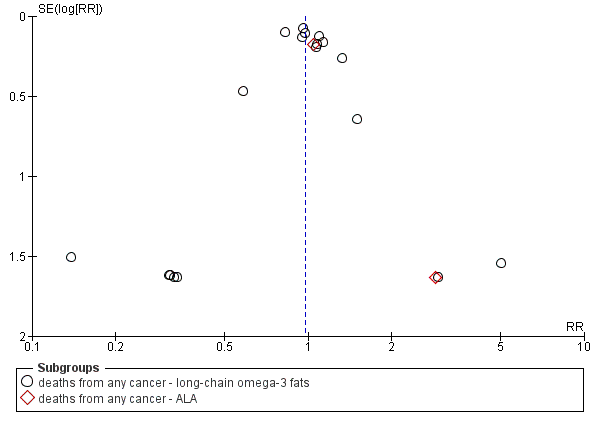


### Supplementary Figure 4. Funnel plot for effects of LCn3 on death from any cancer.


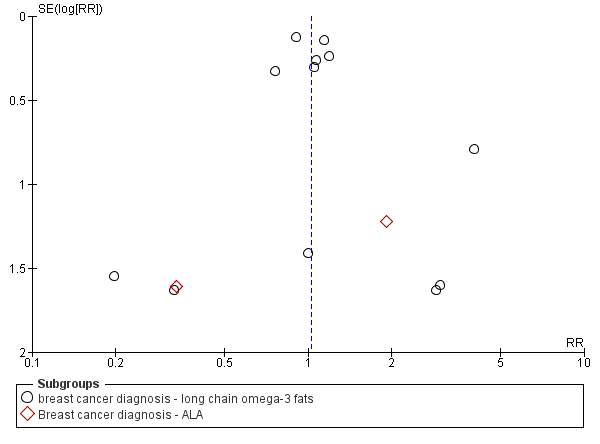


### Supplementary Figure 5. Funnel plot for effects of LCn3 on diagnosis of breast cancer.


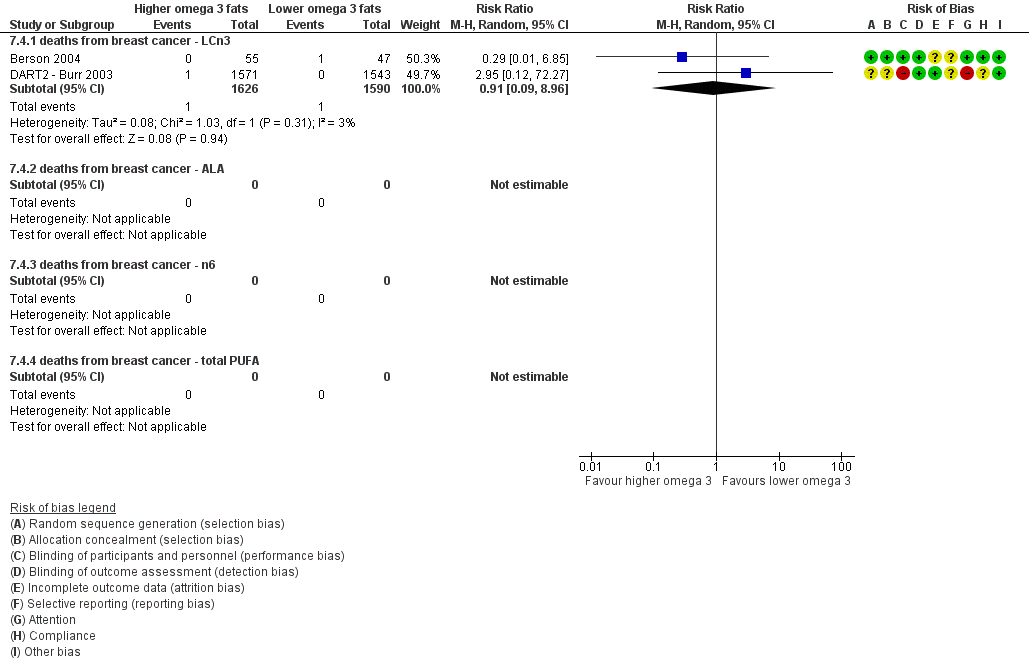


### Supplementary Figure 6. Forest plot showing effects of increasing omega-3, omega-6 and total PUFA on deaths from breast cancer in women participants, using random-effects meta-analyses.


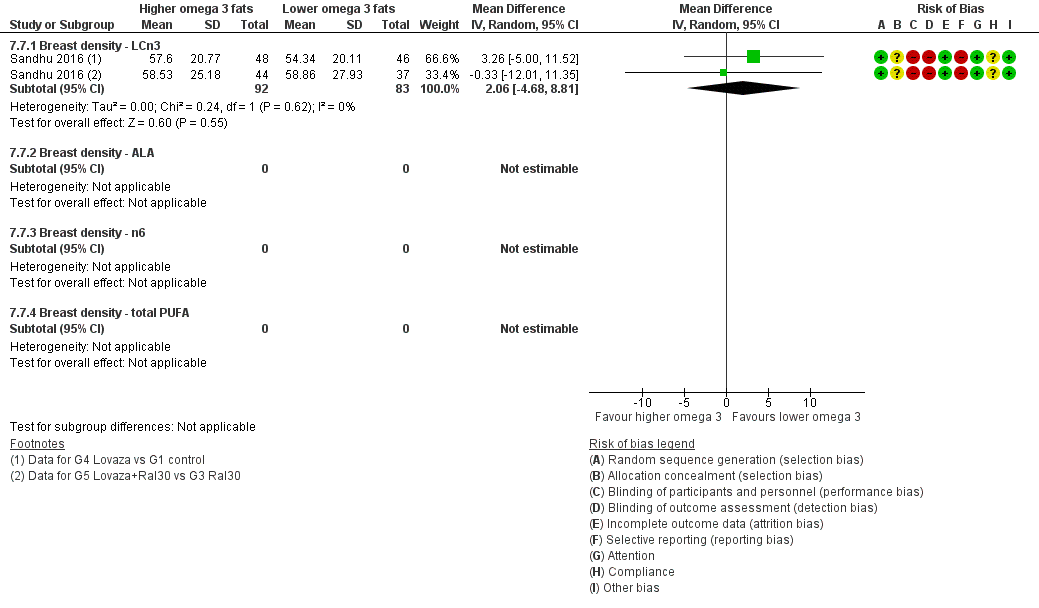


### Supplementary Figure 7. Forest plot showing effects of increasing omega-3, omega-6 and total PUFA on breast density in cm^2^, using random-effects meta-analyses.


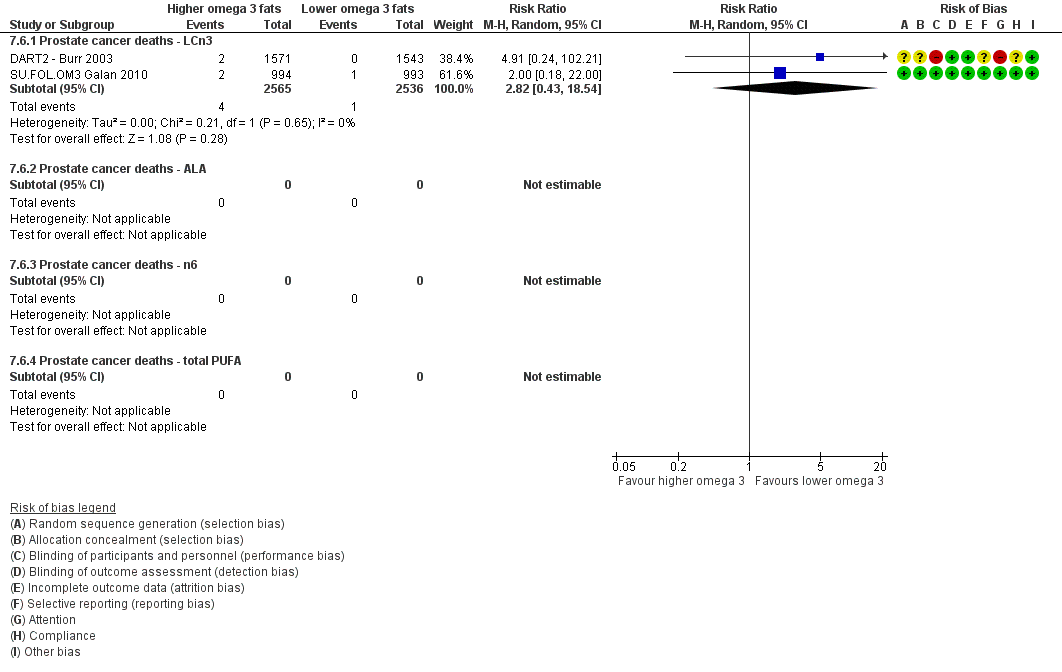


### Supplementary Figure 8. Forest plot showing effects of increasing omega-3, omega-6 and total PUFA on deaths from prostate cancer in male participants, using random-effects meta-analyses.


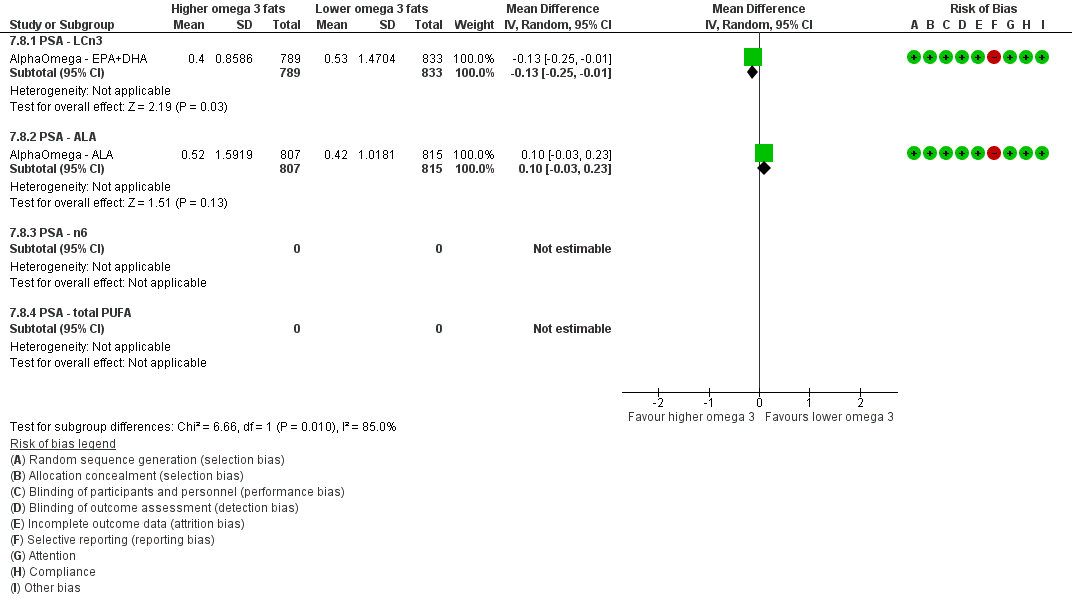


### Supplementary Figure 9. Forest plot showing effects of increasing omega-3, omega-6 and total PUFA on prostate specific antigen (PSA, ng/ml), using random-effects meta-analyses.


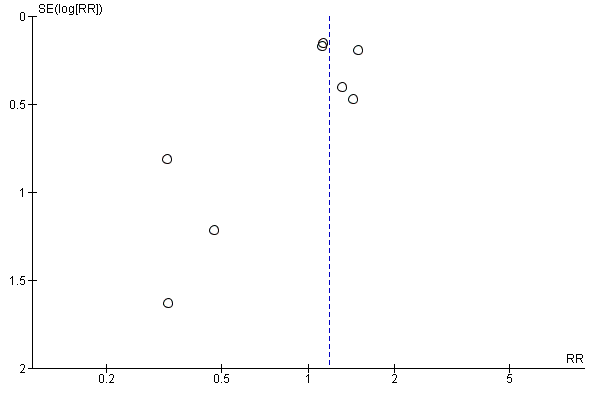


### Supplementary Figure 10. Funnel plot for effects of total PUFA on diagnosis of any cancer.


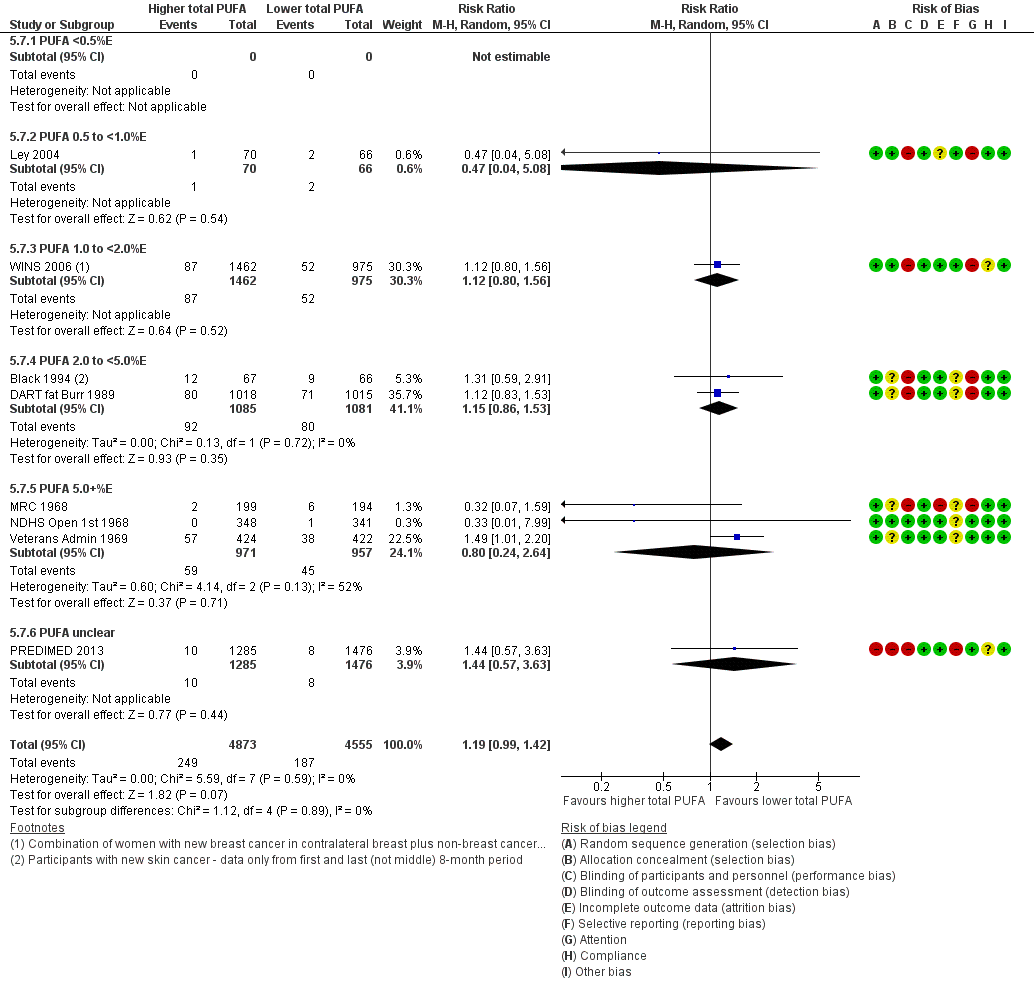


### Supplementary Figure 11. Meta-analysis assessing effects of increasing total PUFA on diagnosis of any cancer, subgrouping by dose of PUFA (as percentage of energy intake).


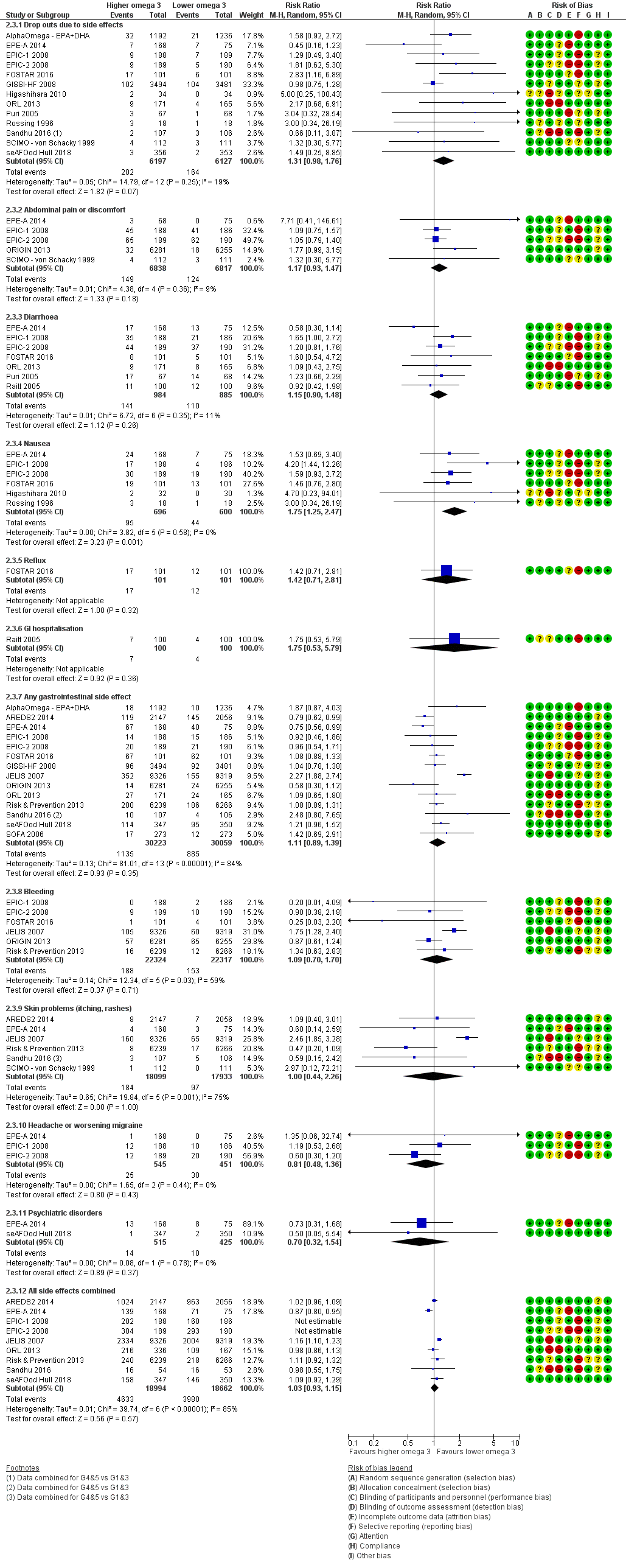


### Supplementary Figure 12. Forest plot showing effects of increasing LCn3 on side effects using random-effects meta-analyses.


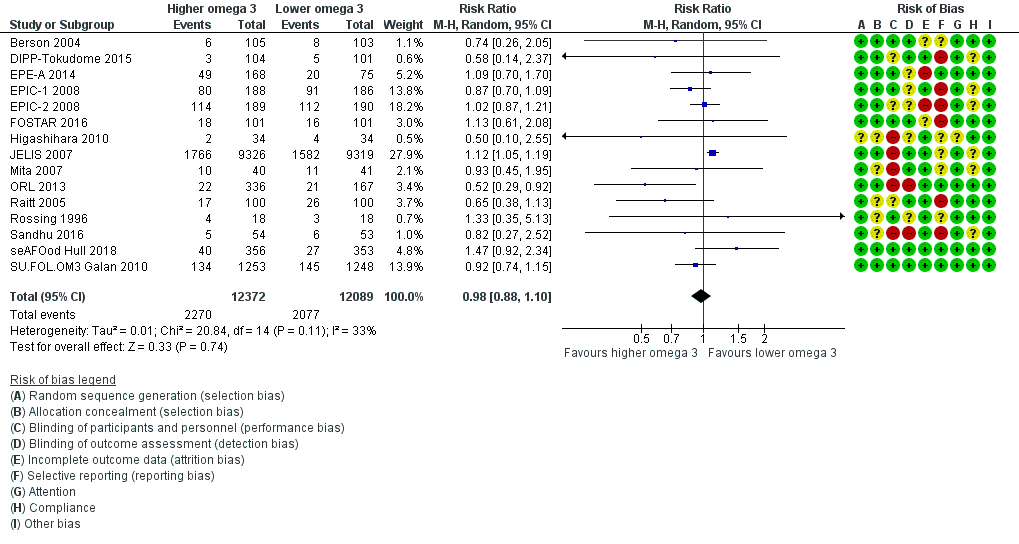


### Supplementary Figure 13. Forest plot showing effects of increasing LCn3 on dropouts using random-effects meta-analyses.


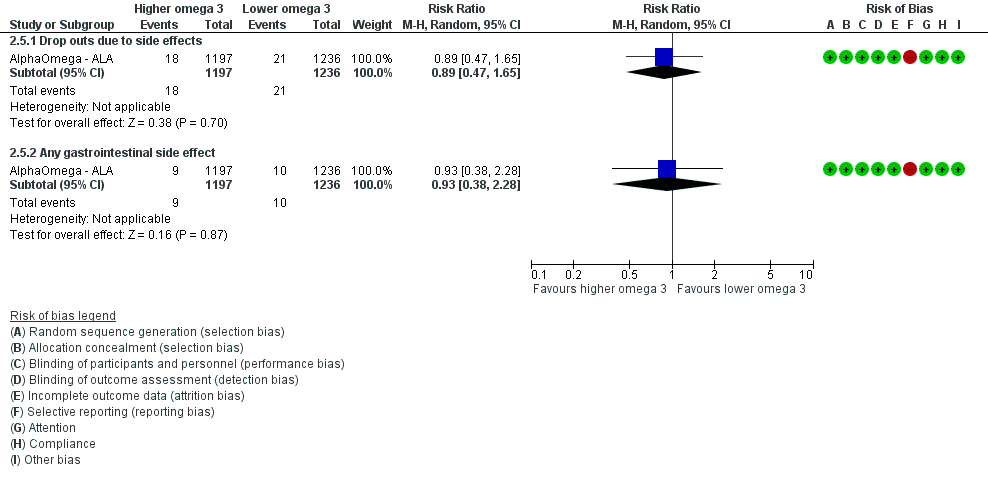


Supplementary Figure 14. Forest plot showing effects of increasing ALA on side effects using random-effects meta-analyses.

**
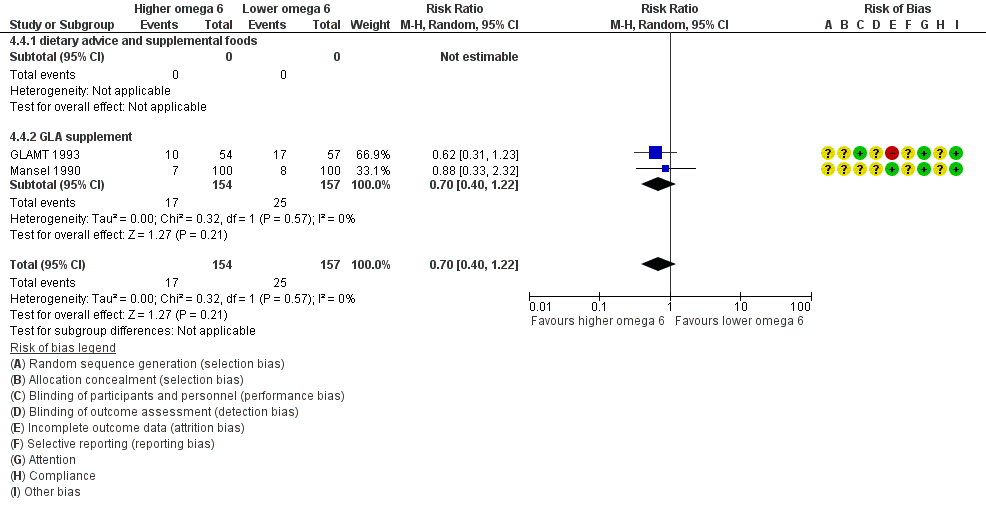
**

### Supplementary Figure 15. Forest plot showing effects of increasing omega-6 on dropouts using random-effects meta-analyses.

## Supplementary Table 1. Table of characteristics, risk of bias and references for included trials

| Trial name & reference | Comparison | Participants | Number randomised | Intervention | Duration of intervention | Summary risk of bias | Location |
| --- | --- | --- | --- | --- | --- | --- | --- |
| AlphaOmega – ALA ^5-8^ | n3 ALA vs MUFA | 60-80 year olds with previous MI | 1197 ALA intervention, 1236 control | Supplementary margarine, 20g/d enriched margarine incorporating 2g/d ALA | 40 months | Low | The Netherlands |
| AlphaOmega - EPA+DHA ^5-8^ | n3 EPA+DHA vs MUFA | 60-80 year olds with previous MI | 1192 EPA/DHA intervention, 1236 control | Supplementary Margarine, 20g/d enriched margarine incorporating 400mg/d LCn3 (240mg/d EPA, 160mg/d DHA) | 40 months | Low | The Netherlands |
| AREDS2 2014 ^9-11^ | n3 EPA+DHA vs nil | 50-85 year olds at high risk of age-related macular degeneration | 2147 DHA/EPA, 2056 placebo | Supplement (capsule), 350 mg/d DHA plus 650 mg/d EPA added to standard AREDS supplement | 60 months | Low | USA |
| ASCEND 2018 ^12, 13^ | n3 EPA + DHA vs MUFA | Patients with DM, without apparent vascular disease | 7740 intervention, 7740 control | Supplement (capsule), 840mg/d EPA+DHA (460mg/d EPA, 380mg/d DHA) as 1 capsule daily | Median 7.4 years | Low | UK |
| Berson 2004 ^14, 15^ | n3 DHA vs n6 LA | People aged 18-55 with retinitis pigmentosa | 221 randomised overall, analysed 105 intervention, 103 control | Supplement (capsule), 1.2g/d DHA plus 1.8g vegetable oil | 48 months | Low | USA |
| Black 1994 ^16, 17^ | Higher vs lower n6, higher vs lower PUFA (inverted) | **People with non-melanoma skin cancer | 66 intervention, 67 control | Dietary advice, reduce total fat to 20%E, including omega 6 and total PUFA | 24 months | Moderate or high | USA |
| DART fat Burr 1989 ^18-20^ | n6 LA vs mixed fats, also higher vs lower PUFA | Men recovering from MI | 1018 Intervention, 1015 control | dietary advice, ↑ PUFA oil & n6 margarines vs usual dietary fats | 24 months | Moderate to high | UK |
| DART fish Burr 1989 ^18-20^ | n3 EPA+DHA vs mixed fat | Men recovering from MI | 1015 intervention, 1018 intervention | Dietary advice, advised to eat ≥2 portions/wk of 200-400g fatty fish, if not possible given MaxEPA capsules, 0.5g EPA/d | 24 months | Moderate or high | UK |
| DART2 - Burr 2003 ^1^ | n3 EPA+DHA vs nil | Men treated for angina | 1571 intervention, 1543 control | dietary advice, advised to eat ≥2 portions/wk of 200-400g fatty fish, if not possible given MaxEPA capsules, 0.5g EPA /d | 3-9 years | Moderate or high | UK |
| DIPP-Tokudome 2015 | n3 EPA+DHA+ALA vs nil | **Patients previously polypectomised for colorectal tumours | 104 intervention, 101 control | Advice plus supplement, reduce total fat intake, decrease n-6 PUFAs, increase fishy n-3 PUFAs, increase n-3 PUFAs from perilla oil rich in ALA, and take 8 capsules of fish oil/day (96 mg/d EPA, 360 mg/d DHA) | 24 months | Moderate or high | Japan |
| DO IT - Einvik 2010 ^21, 22^ | n3 DHA+EPA vs n6 LA | Elderly men with long standing dyslipidaemia or hypertension | 282 intervention, 281 control | Supplement (capsule), 2.4g/d of omega 3 (0.84g/d EPA & 0.48g/d DHA) | 36 months | Moderate or high | Norway |
| EPE-A Sanyal 2014 ^23^ | n3 EPA, low dose vs high dose vs unclear placebo | People with non-alcoholic steatohepatitis or fatty liver disease | 86 intervention (high dose), 82 intervention (low dose), 75 control | Supplement (capsule), High dose EPA-E 2.7g/d, low dose 1.8g/d | 12 months | Moderate or high | USA |
| EPIC-1 2008 ^24^ | n3 EPA+DHA vs mixed fat | Adults with quiescent Crohn’s disease (CDAI) score <150 | 188 intervention, 186 control | Supplement (capsule), 2.2g/d EPA, 0.8g/d DHA | 12 months | Moderate or high | Canada, Europe, Israel, USA |
| EPIC-2 2008 ^24^ | n3 EPA+DHA vs mixed fat | Adults with Crohn’s disease | 189 intervention, 190 control | Supplement (capsule), 2.2g/d EPA, 0.8g/d DHA | 13 months | Moderate or high | Canada, Europe, Israel, USA |
| FOSTAR 2016 ^25^ | n3 EPA+DHA vs low n3 EPA+DHA+ALA | Adults aged 40+ with knee osteoarthritis | 101 intervention, 101 control | Supplementary food (enriched orange juice), 4.5g/d EPA+DHA | 24 months | Low | Australia |
| GISSI-HF 2008 ^26, 27^ | n3 EPA+DHA vs MUFA | Patients with chronic heart failure | 3494 intervention, 3481control | Supplement (capsule), 866mg/d EPA, 1039mg/d DHA, Total Omega-3 Fat: 1905 mg/d | 45 months | Moderate or high | Italy |
| GISSI-P 1999 ^28^ | n3 EPA+DHA vs nil | People with recent MI | 5666 intervention, 5658 control | Supplement (capsule), 850-882 mg/d EPA + DHA daily, ratio 1:2 | 42 months | Moderate or high | Italy |
| GLAMT 1993  ^29^ | n6 GLA vs non-fat | People with mild diabetic neuropathy | 54 intervention, 57 control | Supplement (capsule), 0.48g/d GLA | 12 months | Moderate or high | UK and Finland |
| HARP- Sacks 1995 ^30^ | n3 EPA+DHA vs MUFA | Patients with coronary heart disease | 41 intervention, 39 control | Supplement (capsule), 6g/d LCn3 | 24 months | Moderate or high | USA |
| Higashihara 2010 ^31^ | n3 EPA vs nil | **Prostate cancer patients with PSA levels <0.2 ng/ml 3 months after prostatectomy | 34 intervention, 34 control | Supplement (capsule), 2.4 g/d EPA | 24 months | Moderate or high | Japan |
| Huang 1996 ^32^ | n3 EPA+DHA vs n6 LA | **People with Dukes A or B adenocarcinoma of colon or rectum or severely dysplastic adenomatoid polyps post-surgery | 17 intervention, 10 control | Supplement (capsules), 4g/d EPA + 2g/d DHA | 12 months | Moderate or high | USA |
| JELIS 2007 ^33^ | n3 EPA vs nil | People with hypercholesterolaemia | 9326 intervention, 9319 control | Supplement (capsule), 1.8g/d EPA | 60 months | Moderate or high | Japan |
| Ley 2004 ^34, 35^ | Higher vs lower PUFA (inverted) | Adults with impaired glucose intolerance or high normal blood glucose | 85 intervention, 90 control | Diet advice, aim reduced fat diet (no specific goal stated), which reduced PUFA | 12 months | Low (dietary advice trial) | New Zealand |
| Macsai 2008 ^36^ | n3 ALA vs MUFA | People with meibomian gland dysfunction | 18 ALA intervention, 20 control | Supplement (capsules), 3.3g/d ALA, 1.14g/d LA | 12 months | Moderate to high | USA |
| Mansel 1990 ^37-39^ | n6 GLA vs non-fat | Women with macroscopic breast cysts | 100 intervention, 100 control | Supplement (capsules), estimated at 0.54g/d GLA | 12 months | Moderate or high | UK |
| McIllmurray 1987 ^40^ | n6 GLA vs "inert placebo" | **People within 1 month of operation to remove Dukes's C colorectal cancer | 25 intervention, 24 control | Supplement (capsules), 3.0g/d GLA | 40 months | Moderate to high | UK |
| Mita 2007 ^41^ | n3 EPA vs nil | Japanese type 2 diabetics | 40 intervention, 41 control | Supplement (capsules), 1.8g/d EPA+DHA | 24 months | Moderate or high | Japan |
| MRC 1968 ^42-44^ | n6 LA vs mixed fats, also higher vs lower PUFA | Men who have survived a MI | 199 intervention, 194 control | Diet advice plus oil supplement, reduce dietary fat to 35g/d fat, add 84g/d soya oil | 48 months | Moderate or high | UK |
| NDHS Open 1st 1968 ^43, 45^ | n6 LA vs mixed fats, also higher vs lower PUFA | Free-living men aged 45-54 years | 829 combined intervention groups, 382 control | Diet provided (bought from a trial shop), saturated fats replaced in shop foods by polyunsaturated fats and oils | 12 months | Low | USA |
| OFAMI - Nilsen 2001 ^46^ | n3 EPA+DHA vs n6 LA | Patients recruited 4-8 days after MI | 150 intervention, 150 control | Supplement (capsules), 3.5g/d EPA+DHA | 24 months | Moderate or high | Norway |
| OMEGA 2009 ^47, 48^ | n3 EPA+DHA vs MUFA | People who have had an acute MI | 1940 intervention, 1911 control | supplement (capsules), 460mg/d EPA and 386mg/d DHA | 12 months | Low | Germany |
| ORIGIN 2013 ^49-51^ | n3 EPA+DHA vs MUFA | People at high risk of CVD with impaired fasting glucose, impaired glucose tolerance or DM | 6319 intervention, 6292 control | supplement (capsule), (465mgEPA + 375mgDHA) EPA+DHA 0.84g/d | 72 months | Low | 40 locations in Europe and the Americas |
| ORL Tatsuno 2013 ^52, 53^ | n3 EPA+DHA high dose vs low dose vs n3 EPA | Japanese adults with hypertriglyceridaemia | 171 intervention (4g TAK), 165 control (2g TAK) | Supplement (capsules), 1.68g/d EPA+DHA | 12 months | Moderate or high | Japan |
| PREDIMED 2013 ^54, 55^ | PUFA vs MUFA | People free of CVD but with DM or at least 3 CVD risk factors | 2454 Med with nuts, 2543 Med with olive oil | Dietary advice and food supplement, Mediterranean dietary advice (both groups) plus 30g/d mixed nuts | 60 months | Moderate to high | Spain |
| Puri 2005 ^56^ | n3 EPA vs non-fat | People with Huntington's Disease | 67 intervention, 68 control | Supplement (capsule), 1.9g/d EPA+DHA | 12 months | Low | UK, USA, Canada, Australia |
| Raitt 2005 ^57^ | n3 EPA+DHA vs MUFA | People with implantable cardioverter defibrillators and recent sustained ventricular tachycardia or ventricular fibrillation | 100 intervention, 100 control | Supplement (capsules), 0.76g/d EPA, 0.54g/d DHA (EPA+DHA 1.3g/d) | 24 months | Moderate or high | USA |
| Risk & Prevention 2013 ^58, 59^ | n3 EPA+DHA vs MUFA | Patients with multiple cardiovascular risk factors | 6244 intervention, 6269 control | Supplement (capsules), 0.86g/d EPA+DHA | 60 months | Moderate or high | Italy |
| Rossing 1996 ^60, 61^ | n3 EPA+DHA vs MUFA | Adults with insulin-dependent DM mellitus, diabetic nephropathy & normal BP | 18 intervention, 18 control | Supplement (capsule), 2g/d EPA, 2.6g/d DHA, 4.6g/d EPA+DHA | 12 months | Moderate or high | Denmark |
| Sandhu 2016  ^62, 63^ | n3 EPA+DHA vs nil, +/- raloxifene | *Healthy postmenopausal women with high breast density detected on routine mammogram screening | 54 & 53 intervention, 53 & 53 control | Supplement (capsules), 1.86g/d EPA, 1.5 g/d DHA | 24 months | Moderate or high | USA |
| SCIMO - von Schacky 1999 ^64-66^ | n3 EPA+DHA vs mixed fats | People with angiographically proven coronary artery disease | 112 intervention, 111 control | Supplement (capsule), 1.03g/d EPA+DHA | 24 months | Low | Germany |
| seAFOod Hull 2018 ^67^ | n3 EPA vs MCT | *Bowel cancer screening patients identified as "high risk" at their 1st colonoscopy | 356 intervention, 353 control | supplement (capsule), 2g/d EPA | 12 months | Low | UK |
| Simon 1997  ^68^ | Higher vs lower PUFA (inverted) | *Women with a high risk of breast cancer | 98 intervention, 96 control | Dietary advice, reduced fat including PUFA vs usual diet | 24 months | Moderate or high | USA |
| SOFA 2006 ^69-72^ | n3 EPA+DHA vs n6 LA | People with previous ventricular arrhythmias & implantable cardioverter defibrillators | 273 intervention, 273 control | supplement (capsule), 464mg/d EPA + 335mg/d DHA and 162mg/d other n-3 PUFA, EPA+DHA 0.8g/d | 12 months | Low | 8 countries in Europe |
| SU.FOL.OM3 Galan 2010 ^73-78^ | n3 EPA+DHA vs non-fat | People with a history of MI, unstable angina or ischemic stroke | 1253 intervention, 1248 control | supplement (capsule), 400mg/d EPA and 200mg/d DHA, EPA+DHA 0.6g/d | 48 months | Low | France |
| THIS DIET 2008 ^79^ | n3 EPA+DHA vs nil | Recent survivors of first MI | 51 intervention, 50 control | Dietary advice, Mediterranean style diet high in n3 (>0.75%E from n3, unclear how much EPA, DHA, ALA) | 24 months | Moderate or high | USA |
| Veterans Admin 1969 ^43, 80, 81^ | n6 LA vs SFA, also higher vs lower PUFA | Men living at the Veterans Administration Centre | 424 intervention, 422 control | diet provided (residential institution), total fat 40%E, 2/3 of SFA replaced by unsaturated fats (from corn, soybean, safflower and cottonseed oils) | Up to 96 months | Moderate or high | USA |
| VITAL 2018 ^82^ | n3 EPA & DHA vs MUFA | Multi-ethnic population of > 25,000 apparently healthy adults without cancer or CVD | 12933 intervention, 12938 control | Supplement (capsules), 465 mg/d EPA, 375 mg/d DHA (EPA + DHA 840mg/d) | median 5.3 years | Low | USA |
| WAHA 2016 ^83-85^ | n3 ALA vs unclear | Middle aged healthy adults | 362 intervention, 346 control | Supplement (food), usual diet & walnuts (15%E, ~5g/d ALA) vs usual diet | 24 months | Moderate to high | Spain & USA |
| WINS 2006 ^86-88^ | Higher vs lower PUFA (inverted) | **Women with localised resected breast cancer | 975 intervention, 1462 control | dietary advice, reduced fat intake (with reduced PUFA) | 60 months | Low (as diet advice trial) | USA |
|  |  |  |  |  |  |  |  |
| Summary:  47 trials,  49 comparisons | 34 LCn3  3 ALA  8 n6  9 total PUFA | 38 Normal cancer risk  3 *Cancer risk factors  6 **Previous cancer | 97,548 LCn3  3,179 ALA  4,976 n6  11,573 tot PUFA  Total: 108,194 |  | Mean 30.4 months | 17 trials at Low summary risk of bias | 15 N America  20 Europe  2 Australia/NZ  5 Japan  5 combined |

|  |  |
| --- | --- |

Footnotes

ALA = alpha-linolenic acid, BP = blood pressure, CVD = cardiovascular disease, DHA = docosahexaenoic acid, DM = diabetes mellitus, DPA = docosapentaenoic acid, E = energy intake, EPA = eicosapentaenoic acid or icosapentaenoic acid, LCn3 = long-chain omega-3, MI = myocardial infarction, MUFA = mono-unsaturated fatty acids, n3 = omega 3, PUFA = polyunsaturated fatty acids, SFA = saturated fatty acids, TG = serum triglycerides.

Colour coding: LCn3 uncoloured, ALA blue, n6 yellow, total PUFA red, N6 and PUFA pink.

## Supplementary Table 2. High vs low LCn3 (primary outcomes)

| **Outcome** | **Sensitivity Analysis (SA) or Subgroup** | **Studies** | **Participants** | **Statistical Method** | **Effect Estimate** | **I^2^, %** | **p-value*** |
| --- | --- | --- | --- | --- | --- | --- | --- |
| All cancer diagnoses | Main | 27 | 113557 | Risk Ratio (M-H, Random, 95% CI) | 1.02 [0.98, 1.07] | 0 | - |
|  | SA Fixed effects M-H | 27 | 113557 | Risk Ratio (M-H, Fixed, 95% CI) | 1.03 [0.98, 1.07] | 0 | - |
|  | SA Fixed effects Peto | 27 | 113557 | Peto Odds Ratio (Peto, Fixed, 95% CI) | 1.03 [0.98, 1.08] | 0 | - |
|  | SA Low summary risk of bias | 12 | 66335 | Risk Ratio (M-H, Random, 95% CI) | 1.01 [0.96, 1.06] | 0 | - |
|  | SA compliance | 12 | 34827 | Risk Ratio (M-H, Random, 95% CI) | 1.03 [0.96, 1.10] | 0 | - |
|  | SA n>100 | 25 | 113440 | Risk Ratio (M-H, Random, 95% CI) | 1.02 [0.98, 1.07] | 0 | - |
|  | Duration: 12 to <24 months duration | 9 | 6464 | Risk Ratio (M-H, Random, 95% CI) | 1.03 [0.91, 1.15] | 0 | 0.96 |
|  | Duration: 24 to <48 months duration | 10 | 15144 | Risk Ratio (M-H, Random, 95% CI) | 1.05 [0.91, 1.21] | 0 |  |
|  | Duration: 48+ months duration | 8 | 91949 | Risk Ratio (M-H, Random, 95% CI) | 1.02 [0.97, 1.07] | 0 |  |
|  | Dose: ≤400mg/d LCn3 | 0 | 0 | Risk Ratio (M-H, Random, 95% CI) | Not estimable | - | 0.93 |
|  | Dose: >400 to ≤1400mg/d LCn3 | 14 | 91676 | Risk Ratio (M-H, Random, 95% CI) | 1.02 [0.98, 1.07] | 0 |  |
|  | Dose: >1400 to ≤2400mg/d LCn3 | 7 | 20599 | Risk Ratio (M-H, Random, 95% CI) | 1.03 [0.93, 1.14] | 0 |  |
|  | Dose: >2400mg/d to ≤4400mg/d LCn3 | 2 | 738 | Risk Ratio (M-H, Random, 95% CI) | 0.99 [0.10, 9.52] | 0 |  |
|  | Dose: >4400mg/d LCn3 | 2 | 238 | Risk Ratio (M-H, Random, 95% CI) | 1.40 [0.64, 3.10] | 0 |  |
|  | Dose: dose unclear | 2 | 306 | Risk Ratio (M-H, Random, 95% CI) | 0.96 [0.75, 1.23] | 0 |  |
|  | LCn3 replacing MUFA | 7 | 70432 | Risk Ratio (M-H, Random, 95% CI) | 1.02 [0.97, 1.07] | 0 | 0.23 |
|  | LCn3 replacing omega-6 | 3 | 1317 | Risk Ratio (M-H, Random, 95% CI) | 0.58 [0.29, 1.17] | 0 |  |
|  | LCn3 replacing SFA | 0 | 0 | Risk Ratio (M-H, Random, 95% CI) | Not estimable | - |  |
|  | LCn3 replacing CHO | 0 | 0 | Risk Ratio (M-H, Random, 95% CI) | Not estimable | - |  |
|  | LCn3 replacing other or unclear | 17 | 41808 | Risk Ratio (M-H, Random, 95% CI) | 1.05 [0.97, 1.13] | 0 |  |
|  | Intervention: dietary advice | 1 | 101 | Risk Ratio (M-H, Random, 95% CI) | 2.94 [0.12, 70.56] | - | 0.80 |
|  | Intervention: supplementary capsules | 23 | 111016 | Risk Ratio (M-H, Random, 95% CI) | 1.02 [0.98, 1.07] | 0 |  |
|  | Intervention: supplemental foods | 1 | 202 | Risk Ratio (M-H, Random, 95% CI) | 1.33 [0.59, 3.02] | - |  |
|  | Intervention: all foods provided | 0 | 0 | Risk Ratio (M-H, Random, 95% CI) | Not estimable | - |  |
|  | Intervention: combination | 2 | 2238 | Risk Ratio (M-H, Random, 95% CI) | 1.10 [0.80, 1.50] | 61 |  |
|  | Baseline cancer risk: low - usual population | 24 | 112499 | Risk Ratio (M-H, Random, 95% CI) | 1.03 [0.98, 1.08] | 0 | 0.81 |
|  | Baseline cancer risk: moderate - CA risk factors | 2 | 853 | Risk Ratio (M-H, Random, 95% CI) | 0.92 [0.43, 1.96] | 11 |  |
|  | Baseline cancer risk: high - previous CA | 1 | 205 | Risk Ratio (M-H, Random, 95% CI) | 0.95 [0.75, 1.22] | - |  |
|  | Mean age <50 years | 6 | 1346 | Risk Ratio (M-H, Random, 95% CI) | 1.04 [0.29, 3.75] | 0 | 0.95 |
|  | Mean age 50 to <65 years | 17 | 80934 | Risk Ratio (M-H, Random, 95% CI) | 1.03 [0.98, 1.08] | 0 |  |
|  | Mean age 65+ years | 4 | 31277 | Risk Ratio (M-H, Random, 95% CI) | 1.01 [0.95, 1.09] | 0 |  |
|  | Men & women mixed | 24 | 110748 | Risk Ratio (M-H, Random, 95% CI) | 1.02 [0.98, 1.07] | 0 | 0.54 |
|  | Men only | 2 | 2596 | Risk Ratio (M-H, Random, 95% CI) | 0.87 [0.35, 2.19] | 77 |  |
|  | Women only | 1 | 213 | Risk Ratio (M-H, Random, 95% CI) | 0.20 [0.01, 4.08] | - |  |
|  |  |  |  |  |  |  |  |
| Cancer deaths | Main | 18 | 99336 | Risk Ratio (M-H, Random, 95% CI) | 0.97 [0.90, 1.06] | 0 | - |
|  | SA fixed effects M-H | 18 | 99336 | Risk Ratio (M-H, Fixed, 95% CI) | 0.97 [0.90, 1.05] | 0 | - |
|  | SA fixed effects Peto | 18 | 99336 | Peto Odds Ratio (Peto, Fixed, 95% CI) | 0.97 [0.89, 1.06] | 7 | - |
|  | SA Low summary risk of bias | 6 | 61433 | Risk Ratio (M-H, Random, 95% CI) | 0.94 [0.86, 1.04] | 0 | - |
|  | SA compliance | 7 | 34122 | Risk Ratio (M-H, Random, 95% CI) | 1.00 [0.85, 1.18] | 0 | - |
|  | SA n>100 | 16 | 99194 | Risk Ratio (M-H, Random, 95% CI) | 0.97 [0.90, 1.06] | 0 | - |
|  | Duration: 12 to <24 months duration | 2 | 742 | Risk Ratio (M-H, Random, 95% CI) | 0.99 [0.10, 9.52] | 0 | 0.88 |
|  | Duration: 24 to <48 months duration | 9 | 26379 | Risk Ratio (M-H, Random, 95% CI) | 1.01 [0.85, 1.20] | 0 |  |
|  | Duration: 48+ months duration | 7 | 72215 | Risk Ratio (M-H, Random, 95% CI) | 0.96 [0.88, 1.05] | 0 |  |
|  | Dose: ≤400mg/d LCn3 | 1 | 4837 | Risk Ratio (M-H, Random, 95% CI) | 1.08 [0.76, 1.53] | - | 0.70 |
|  | Dose: >400 to ≤1400mg/d LCn3 | 10 | 86135 | Risk Ratio (M-H, Random, 95% CI) | 0.97 [0.89, 1.06] | 0 |  |
|  | Dose: >1400 to ≤2400mg/d LCn3 | 2 | 7037 | Risk Ratio (M-H, Random, 95% CI) | 0.94 [0.73, 1.23] | 0 |  |
|  | Dose: >2400 to ≤4400mg/d LCn3 | 3 | 1042 | Risk Ratio (M-H, Random, 95% CI) | 1.77 [0.29, 10.83] | 0 |  |
|  | Dose: >4400mg/d LCn3 | 1 | 80 | Risk Ratio (M-H, Random, 95% CI) | 0.32 [0.01, 7.57] | - |  |
|  | Dose: unclear | 1 | 205 | Risk Ratio (M-H, Random, 95% CI) | 0.14 [0.01, 2.65] | - |  |
|  | LCn3 replacing MUFA | 7 | 78284 | Risk Ratio (M-H, Random, 95% CI) | 0.95 [0.87, 1.04] | 0 | 0.25 |
|  | LCn3 replacing omega-6 | 3 | 1071 | Risk Ratio (M-H, Random, 95% CI) | 0.66 [0.28, 1.56] | 0 |  |
|  | LCn3 replacing SFA | 0 | 0 | Risk Ratio (M-H, Random, 95% CI) | Not estimable | - |  |
|  | LCn3 replacing CHO | 0 | 0 | Risk Ratio (M-H, Random, 95% CI) | Not estimable | - |  |
|  | LCn3 replacing other or unclear | 8 | 19981 | Risk Ratio (M-H, Random, 95% CI) | 1.13 [0.91, 1.41] | 0 |  |
|  | Intervention: dietary advice | 0 | 0 | Risk Ratio (M-H, Random, 95% CI) | Not estimable | - | 0.76 |
|  | Intervention: supplementary capsules | 14 | 89147 | Risk Ratio (M-H, Random, 95% CI) | 0.96 [0.88, 1.05] | 0 |  |
|  | Intervention: supplemental foods | 1 | 4837 | Risk Ratio (M-H, Random, 95% CI) | 1.08 [0.76, 1.53] | - |  |
|  | Intervention: all foods provided | 0 | 0 | Risk Ratio (M-H, Random, 95% CI) | Not estimable | - |  |
|  | Intervention: combination | 3 | 5352 | Risk Ratio (M-H, Random, 95% CI) | 1.06 [0.67, 1.68] | 6 |  |
|  | Baseline cancer risk: low - usual population | 16 | 99069 | Risk Ratio (M-H, Random, 95% CI) | 0.97 [0.90, 1.06] | 0 | 0.15 |
|  | Baseline cancer risk: moderate - CA risk factors | 0 | 0 | Risk Ratio (M-H, Random, 95% CI) | Not estimable | - |  |
|  | Baseline cancer risk: high - previous CA | 2 | 267 | Risk Ratio (M-H, Random, 95% CI) | 0.20 [0.02, 1.75] | 0 |  |
|  | Mean age <50 | 3 | 950 | Risk Ratio (M-H, Random, 95% CI) | 0.69 [0.11, 4.33] | 0 | 0.93 |
|  | Mean age 50-<65 | 11 | 60140 | Risk Ratio (M-H, Random, 95% CI) | 0.98 [0.88, 1.08] | 2 |  |
|  | Mean age 65+ | 4 | 38246 | Risk Ratio (M-H, Random, 95% CI) | 0.97 [0.84, 1.12] | 0 |  |
|  | Men & women mixed | 14 | 93564 | Risk Ratio (M-H, Random, 95% CI) | 0.97 [0.89, 1.06] | 0 | 0.92 |
|  | Men only | 4 | 5772 | Risk Ratio (M-H, Random, 95% CI) | 0.99 [0.70, 1.40] | 0 |  |
|  | Women only | 0 | 0 | Risk Ratio (M-H, Random, 95% CI) | Not estimable | - |  |
|  |  |  |  |  |  |  |  |
| Breast cancer diagnoses | Main | 12 | 44295 | Risk Ratio (M-H, Random, 95% CI) | 1.03 [0.89, 1.20] | 0 | - |
|  | SA fixed effects M-H | 12 | 44295 | Risk Ratio (M-H, Fixed, 95% CI) | 1.03 [0.89, 1.20] | 0 | - |
|  | SA fixed effects Peto | 12 | 44295 | Peto Odds Ratio (Peto, Fixed, 95% CI) | 1.03 [0.89, 1.21] | 6 | - |
|  | SA Low summary risk of bias | 7 | 26371 | Risk Ratio (M-H, Random, 95% CI) | 1.02 [0.87, 1.22] | 0 | - |
|  | SA compliance | 6 | 13908 | Risk Ratio (M-H, Random, 95% CI) | 0.96 [0.72, 1.30] | 1 | - |
|  | SA n >100 | 11 | 44285 | Risk Ratio (M-H, Random, 95% CI) | 1.03 [0.88, 1.20] | 0 | - |
|  | Duration: 12 to <24 months duration | 2 | 107 | Risk Ratio (M-H, Random, 95% CI) | 2.92 [0.33, 25.76] | 0 | 0.41 |
|  | Duration: 24 to <48 months duration | 2 | 313 | Risk Ratio (M-H, Random, 95% CI) | 0.39 [0.05, 2.94] | 0 |  |
|  | Duration: 48+ months duration | 8 | 43875 | Risk Ratio (M-H, Random, 95% CI) | 1.03 [0.89, 1.20] | 0 |  |
|  | Dose: ≤400mg/d LCn3 | 0 | 0 | Risk Ratio (M-H, Random, 95% CI) | Not estimable | - | 0.60 |
|  | Dose: >400 to ≤1400mg/d LCn3 | 7 | 31089 | Risk Ratio (M-H, Random, 95% CI) | 1.05 [0.90, 1.23] | 0 |  |
|  | Dose: >1400 to ≤2400mg/d LCn3 | 3 | 13096 | Risk Ratio (M-H, Random, 95% CI) | 0.76 [0.41, 1.42] | 0 |  |
|  | Dose: >2400 to ≤4400mg/d LCn3 | 0 | 0 | Risk Ratio (M-H, Random, 95% CI) | Not estimable | - |  |
|  | Dose: >4400mg/d LCn3 | 2 | 110 | Risk Ratio (M-H, Random, 95% CI) | 1.32 [0.18, 10.01] | 0 |  |
|  | Dose: unclear | 0 | 0 | Risk Ratio (M-H, Random, 95% CI) | Not estimable | - |  |
|  | LCn3 replacing MUFA | 5 | 28095 | Risk Ratio (M-H, Random, 95% CI) | 1.04 [0.89, 1.23] | 0 | 0.71 |
|  | LCn3 replacing omega-6 | 1 | 102 | Risk Ratio (M-H, Random, 95% CI) | 0.29 [0.01, 6.85] | - |  |
|  | LCn3 replacing SFA | 0 | 0 | Risk Ratio (M-H, Random, 95% CI) | Not estimable | - |  |
|  | LCn3 replacing CHO | 0 | 0 | Risk Ratio (M-H, Random, 95% CI) | Not estimable | - |  |
|  | LCn3 replacing other or unclear | 6 | 16098 | Risk Ratio (M-H, Random, 95% CI) | 0.99 [0.63, 1.54] | 5 |  |
|  | Intervention: dietary advice | 0 | 0 | Risk Ratio (M-H, Random, 95% CI) | Not estimable | - | 0.76 |
|  | Intervention: supplementary capsules | 11 | 44195 | Risk Ratio (M-H, Random, 95% CI) | 1.03 [0.89, 1.20] | 0 |  |
|  | Intervention: supplemental foods | 1 | 100 | Risk Ratio (M-H, Random, 95% CI) | 0.67 [0.04, 10.35] | - |  |
|  | Intervention: all foods provided | 0 | 0 | Risk Ratio (M-H, Random, 95% CI) | Not estimable | - |  |
|  | Baseline cancer risk: low - usual population | 11 | 44082 | Risk Ratio (M-H, Random, 95% CI) | 1.04 [0.89, 1.20] | 0 | 0.28 |
|  | Baseline cancer risk: moderate - CA risk factors | 1 | 213 | Risk Ratio (M-H, Random, 95% CI) | 0.20 [0.01, 4.08] | - |  |
|  | Baseline cancer risk: high - previous CA | 0 | 0 | Risk Ratio (M-H, Random, 95% CI) | Not estimable | - |  |
|  | Mean age <50 | 2 | 112 | Risk Ratio (M-H, Random, 95% CI) | 0.99 [0.10, 9.88] | 11 | 0.43 |
|  | Mean age 50-<65 | 8 | 28710 | Risk Ratio (M-H, Random, 95% CI) | 1.13 [0.92, 1.38] | 0 |  |
|  | Mean age 65+ | 2 | 15473 | Risk Ratio (M-H, Random, 95% CI) | 0.92 [0.73, 1.16] | 0 |  |
|  | Men & women mixed | 11 | 44082 | Risk Ratio (M-H, Random, 95% CI) | 1.04 [0.89, 1.20] | 0 | 0.28 |
|  | Men only | 0 | 0 | Risk Ratio (M-H, Random, 95% CI) | Not estimable | - |  |
|  | Women only | 1 | 213 | Risk Ratio (M-H, Random, 95% CI) | 0.20 [0.01, 4.08] | - |  |
|  |  |  |  |  |  |  |  |
| Breast cancer deaths | Main | 2 | 3216 | Risk Ratio (M-H, Random, 95% CI) | 0.91 [0.09, 8.96] | 3 | - |
|  | SA fixed effects M-H | 2 | 3216 | Risk Ratio (M-H, Fixed, 95% CI) | 0.92 [0.13, 6.26] | 3 | - |
|  | SA fixed effects Peto | 2 | 3216 | Peto Odds Ratio (Peto, Fixed, 95% CI) | 0.92 [0.06, 14.71] | 53 | - |
|  | SA low summary RoB | 1 | 102 | Risk Ratio (M-H, Random, 95% CI) | 0.29 [0.01, 6.85] | - | - |
|  | SA compliance | 1 | 102 | Risk Ratio (M-H, Random, 95% CI) | 0.29 [0.01, 6.85] | - | - |
|  | SA n >100 | 2 | 3216 | Risk Ratio (M-H, Random, 95% CI) | 0.91 [0.09, 8.96] | 3 | - |
|  |  |  |  |  |  |  |  |
| Prostate cancer diagnoses | Main | 7 | 38525 | Risk Ratio (M-H, Random, 95% CI) | 1.10 [0.97, 1.24] | 0 | - |
|  | SA fixed effects M-H | 7 | 38525 | Risk Ratio (M-H, Fixed, 95% CI) | 1.10 [0.98, 1.24] | 0 | - |
|  | SA fixed effects Peto | 7 | 38525 | Peto Odds Ratio (Peto, Fixed, 95% CI) | 1.10 [0.97, 1.25] | 0 | - |
|  | SA Low summary risk of bias | 6 | 36492 | Risk Ratio (M-H, Random, 95% CI) | 1.10 [0.98, 1.25] | 0 | - |
|  | SA compliance | 4 | 18658 | Risk Ratio (M-H, Random, 95% CI) | 1.17 [0.99, 1.39] | 0 | - |
|  | SA n >100 | 7 | 38525 | Risk Ratio (M-H, Random, 95% CI) | 1.10 [0.97, 1.24] | 0 | - |
|  |  |  |  |  |  |  |  |
| Prostate cancer deaths | Main | 2 | 5101 | Risk Ratio (M-H, Random, 95% CI) | 2.82 [0.43, 18.54] | 0 | - |
|  | SA fixed effects M-H | 2 | 5101 | Risk Ratio (M-H, Fixed, 95% CI) | 2.97 [0.47, 18.89] | 0 | - |
|  | SA fixed effects Peto | 2 | 5101 | Peto Odds Ratio (Peto, Fixed, 95% CI) | 3.30 [0.57, 19.05] | 0 | - |
|  | SA low summary risk of bias | 1 | 1987 | Risk Ratio (M-H, Random, 95% CI) | 2.00 [0.18, 22.00] | - | - |
|  | SA compliance | 1 | 1987 | Risk Ratio (M-H, Random, 95% CI) | 2.00 [0.18, 22.00] | - | - |
|  | SA n >100 | 2 | 5101 | Risk Ratio (M-H, Random, 95% CI) | 2.82 [0.43, 18.54] | 0 | - |
|  |  |  |  |  |  |  |  |
| Dichotomous markers of cancer risk | PSA >2ng/ml twice at consecutive measurements | 1 | 62 | Risk Ratio (M-H, Fixed, 95% CI) | 0.47 [0.16, 1.40] | - | - |
|  |  |  |  |  |  |  |  |
| Continuous markers of cancer risk | Breast density LCn3, cm^2^ | 1 | 175 | Mean Difference (IV, Random, 95% CI) | 2.06 [-4.68, 8.81] | - | - |
|  | PSA, ng/ml | 1 | 1622 | Mean Difference (IV, Random, 95% CI) | -0.13 [-0.25, -0.01] | - | - |

* test for subgroup differences, p-value

## Supplementary Table 3. High vs low LCn3 (secondary outcomes)

| **Outcome** | **Sensitivity Analysis (SA) or Subgroup** | **Studies** | **Participants** | **Statistical Method** | **Effect Estimate** | **I^2^, %** |
| --- | --- | --- | --- | --- | --- | --- |
| Quality of life | - | 0 | 0 | Mean Difference (IV, Random, 95% CI) | Not estimable | - |
| Adiposity, Weight or BMI | Weight, kg | 3 | 14913 | Mean Difference (IV, Random, 95% CI) | 0.42 [-0.87, 1.71] | 63 |
|  | BMI, kg/m2 | 4 | 14268 | Mean Difference (IV, Random, 95% CI) | 0.06 [-0.08, 0.19] | 0 |
|  | Waist circumference, cm | 1 | 71 | Mean Difference (IV, Random, 95% CI) | -1.40 [-7.94, 5.14] | - |
| Side effects | Drop outs due to side effects | 13 | 12324 | Risk Ratio (M-H, Random, 95% CI) | 1.31 [0.98, 1.76] | 19 |
|  | Abdominal pain or discomfort | 5 | 13655 | Risk Ratio (M-H, Random, 95% CI) | 1.17 [0.93, 1.47] | 9 |
|  | Diarrhoea | 7 | 1869 | Risk Ratio (M-H, Random, 95% CI) | 1.15 [0.90, 1.48] | 11 |
|  | Nausea | 6 | 1296 | Risk Ratio (M-H, Random, 95% CI) | 1.75 [1.25, 2.47] | 0 |
|  | Any gastrointestinal side effect | 14 | 60282 | Risk Ratio (M-H, Random, 95% CI) | 1.11 [0.89, 1.39] | 84 |
|  | Bleeding | 6 | 44641 | Risk Ratio (M-H, Random, 95% CI) | 1.09 [0.70, 1.70] | 59 |
|  | Skin problems (itching, rashes) | 6 | 36032 | Risk Ratio (M-H, Random, 95% CI) | 1.00 [0.44, 2.26] | 75 |
|  | Headache or worsening migraine | 3 | 996 | Risk Ratio (M-H, Random, 95% CI) | 0.81 [0.48, 1.36] | 0 |
|  | Psychiatric disorders | 2 | 940 | Risk Ratio (M-H, Random, 95% CI) | 0.70 [0.32, 1.54] | 0 |
|  | All side effects combined | 9 | 37656 | Risk Ratio (M-H, Random, 95% CI) | 1.03 [0.93, 1.15] | 85 |
| Drop outs |  | 15 | 24461 | Risk Ratio (M-H, Random, 95% CI) | 0.98 [0.88, 1.10] | 33 |

## Supplementary Table 4. GRADE table: summary of findings of effects of omega-3 fats (LCn3 and ALA) on cancers

| **High compared to low omega 3 (LCn3 and ALA) for cancers** | | | | | | |
| --- | --- | --- | --- | --- | --- | --- |
| **Patient or population**: adults, **Setting**: community, **Intervention**: Higher omega-3 intake, **Comparison**: lower omega-3 intake | | | | | | |
| Outcomes | **Anticipated absolute effects^*^** (95% CI) | | Relative effect (95% CI) | № of participants  (studies) | Certainty of the evidence (GRADE) | Comments |
|  | **Risk with low omega 3 (primary outcomes)** | **Risk with High** |  |  |  |  |
| Cancer diagnoses - LCn3 | 64 per 1,000 | **65 per 1,000** (63 to 68) | **RR 1.02** (0.98 to 1.07) | 113557 (27 RCTs) | ⨁⨁⨁⨁ HIGH | Increasing LCn3 has little or no effect on risk of diagnosis of any cancer. |
| Cancer deaths - LCn3 | 23 per 1,000 | **23 per 1,000** (21 to 25) | **RR 0.97** (0.90 to 1.06) | 99336 (18 RCTs) | ⨁⨁⨁◯ MODERATE ^a^ | Increasing LCn3 probably has little or no effect on risk of cancer death. |
| Breast cancer diagnoses - LCn3 | 15 per 1,000 | **15 per 1,000** (13 to 18) | **RR 1.03** (0.89 to 1.20) | 44295 (12 RCTs) | ⨁⨁⨁◯ MODERATE ^b,c^ | Increasing LCn3 probably has little or no effect on risk of breast cancer diagnosis. |
| Breast cancer deaths - LCn3 | 1 per 1,000 | **1 per 1,000** (0 to 6) | **RR 0.91** (0.09 to 8.96) | 3216 (2 RCTs) | ⨁◯◯◯ VERY LOW ^d,e^ | The effect of increasing LCn3 on breast cancer deaths is unclear as the evidence is of very low quality. |
| Prostate cancer diagnoses - LCn3 | 25 per 1,000 | **28 per 1,000** (24 to 31) | **RR 1.10** (0.97 to 1.24) | 38525 (7 RCTs) | ⨁⨁◯◯ LOW ^f,g^ | Increasing LCn3 may increase the risk of prostate cancer. |
| Prostate cancer deaths - LCn3 | 0 per 1,000 | **1 per 1,000** (0 to 7) | **RR 2.82** (0.43 to 18.54) | 5101 (2 RCTs) | ⨁◯◯◯ VERY LOW ^e,f^ | The effect of increasing LCn3 on prostate cancer death is unclear as the evidence is of very low quality. |
| Cancer diagnoses - ALA | 22 per 1,000 | **21 per 1,000** (8 to 55) | **RR 0.98** (0.38 to 2.55) | 752 (2 RCTs) | ⨁◯◯◯ VERY LOW ^e,h^ | The effect of increasing ALA on diagnosis of any cancer is unclear as the evidence was of very low quality. |
| Cancer deaths - ALA | 22 per 1,000 | **23 per 1,000** (16 to 32) | **RR 1.05** (0.74 to 1.49) | 5545 (2 RCTs) | ⨁⨁⨁◯ MODERATE ^c^ | Increasing ALA probably has little or no effect on risk of cancer death. |
| Breast cancer diagnoses - ALA | 8 per 1,000 | **9 per 1,000** (1 to 58) | **RR 1.11** (0.17 to 7.40) | 513 (2 RCTs) | ⨁◯◯◯ VERY LOW ^e,h,k^ | The effect of increasing ALA on risk of breast cancer diagnosis is unclear as the evidence is of very low quality. |
| Breast cancer deaths - ALA | not pooled | not pooled | not pooled | (0 RCTs) | - | We found no evidence to address this issue. |
| Prostate cancer diagnoses - ALA | 10 per 1,000 | **13 per 1,000** (7 to 23) | **RR 1.30** (0.72 to 2.32) | 4010 (2 RCTs) | ⨁⨁◯◯ LOW ^i,j^ | Increasing ALA may increase the risk of prostate cancer diagnosis. |
| Prostate cancer deaths - ALA | not pooled | not pooled | not pooled | (0 RCTs) | - | No evidence found |
| ***The risk in the intervention group** (and its 95% confidence interval) is based on the assumed risk in the comparison group and the **relative effect** of the intervention (and its 95% CI).  **CI:** Confidence interval; **RR:** Risk ratio | | | | | | |
| **GRADE Working Group grades of evidence** **High certainty:** We are very confident that the true effect lies close to that of the estimate of the effect **Moderate certainty:** We are moderately confident in the effect estimate: The true effect is likely to be close to the estimate of the effect, but there is a possibility that it is substantially different **Low certainty:** Our confidence in the effect estimate is limited: The true effect may be substantially different from the estimate of the effect **Very low certainty:** We have very little confidence in the effect estimate: The true effect is likely to be substantially different from the estimate of effect | | | | | | |

#### Explanations

a. Imprecision: 95% CI included a small reduction in risk as well as little or no effect. Downgraded once.

b. Inconsistency: data were consistent across all sensitivity analyses, including limiting analysis to only trials at low summary risk of bias, and consistent with the suggestion of little or no effect for breast density. Not downgraded.

c. Imprecision: 95% CI included both increases and reductions in risk. Downgraded once.

d. Risk of bias: sensitivity analysis retaining only trials at low summary risk of bias altered apparent effect. Downgraded once.

e. Imprecision: 95% CI included both important benefit and important harm. Downgraded twice.

f. Inconsistency: While data on prostate cancer diagnosis and deaths across sensitivity analyses are consistent in suggesting that increasing LCn3 increases prostate cancer risk, including limiting to trials at low summary risk of bias, PSA data suggest that LCn3 reduces PSA (which would tend to protect against prostate cancer). Downgraded once.

g. Imprecision: 95% CI included no effect as well as harm. Downgraded once.

h. Risk of bias: Neither included trial was at low summary risk of bias. Downgraded once.

i. Inconsistency: consistent across all sensitivity analyses, including when limiting only to trials at low summary risk of bias, and consistent with PSA data. Not downgraded.

j. Imprecision: 95% CI included benefits as well as harms. Downgraded twice.

k. Inconsistency: interpretation of effects differ according to analysis. Downgraded once.

## Supplementary Table 5. High vs low ALA (primary outcomes)

| **Outcome** | **Sensitivity Analysis (SA) or Subgroup** | **Studies** | **Participants** | **Statistical Method** | **Effect Estimate** | **I^2^, %** |
| --- | --- | --- | --- | --- | --- | --- |
| All cancer diagnoses | Main | 2 | 752 | Risk Ratio (M-H, Random, 95% CI) | 0.98 [0.38, 2.55] | 0 |
|  | SA Fixed effects M-H | 2 | 752 | Risk Ratio (M-H, Fixed, 95% CI) | 0.96 [0.37, 2.46] | 0 |
|  | SA Fixed effects Peto | 2 | 752 | Peto Odds Ratio (Peto, Fixed, 95% CI) | 0.96 [0.36, 2.58] | 2 |
|  | SA Low summary risk of bias | 0 | 0 | Risk Ratio (M-H, Random, 95% CI) | Not estimable | - |
|  | SA compliance | 1 | 708 | Risk Ratio (M-H, Random, 95% CI) | 1.09 [0.40, 2.98] | - |
|  | SA n>100 | 1 | 708 | Risk Ratio (M-H, Random, 95% CI) | 1.09 [0.40, 2.98] | - |
|  |  |  |  |  |  |  |
| Deaths from any cancer | Main | 2 | 5545 | Risk Ratio (M-H, Random, 95% CI) | 1.05 [0.74, 1.49] | 0 |
|  | SA fixed effects M-H | 2 | 5545 | Risk Ratio (M-H, Fixed, 95% CI) | 1.06 [0.75, 1.50] | 0 |
|  | SA fixed effects Peto | 2 | 5545 | Peto Odds Ratio (Peto, Fixed, 95% CI) | 1.06 [0.74, 1.52] | 0 |
|  | SA low summary risk of bias | 1 | 4837 | Risk Ratio (M-H, Random, 95% CI) | 1.04 [0.73, 1.48] | - |
|  | SA compliance | 2 | 5545 | Risk Ratio (M-H, Random, 95% CI) | 1.05 [0.74, 1.49] | 0 |
|  | SA n>100 | 2 | 5545 | Risk Ratio (M-H, Random, 95% CI) | 1.05 [0.74, 1.49] | 0 |
|  |  |  |  |  |  |  |
| Breast cancer diagnoses | Main | 2 | 513 | Risk Ratio (M-H, Random, 95% CI) | 1.11 [0.17, 7.40] | 0 |
|  | SA fixed effects M-H | 2 | 513 | Risk Ratio (M-H, Fixed, 95% CI) | 1.08 [0.18, 6.40] | 0 |
|  | SA fixed effects Peto | 2 | 513 | Peto Odds Ratio (Peto, Fixed, 95% CI) | 1.04 [0.15, 7.45] | 8 |
|  | SA Low summary risk of bias | 0 | 0 | Risk Ratio (M-H, Random, 95% CI) | Not estimable | - |
|  | SA compliance | 1 | 481 | Risk Ratio (M-H, Random, 95% CI) | 1.94 [0.18, 21.28] | - |
|  | SA n>100 | 1 | 481 | Risk Ratio (M-H, Random, 95% CI) | 1.94 [0.18, 21.28] | - |
|  |  |  |  |  |  |  |
| Breast cancer deaths | Main | 0 | 0 | Risk Ratio (M-H, Random, 95% CI) | Not estimable | - |
|  |  |  |  |  |  |  |
| Prostate cancer diagnoses | Main | 2 | 4010 | Risk Ratio (M-H, Random, 95% CI) | 1.30 [0.72, 2.32] | 0 |
|  | SA fixed effects  M-H | 2 | 4010 | Risk Ratio (M-H, Fixed, 95% CI) | 1.31 [0.73, 2.34] | 0 |
|  | SA fixed effects Peto | 2 | 4010 | Peto Odds Ratio (Peto, Fixed, 95% CI) | 1.31 [0.73, 2.34] | 0 |
|  | SA Low summary risk of bias | 1 | 3783 | Risk Ratio (M-H, Random, 95% CI) | 1.23 [0.67, 2.24] | 0 |
|  | SA compliance | 2 | 4010 | Risk Ratio (M-H, Random, 95% CI) | 1.30 [0.72, 2.32] | 0 |
|  | SA n >100 | 2 | 4010 | Risk Ratio (M-H, Random, 95% CI) | 1.30 [0.72, 2.32] | 0 |
|  |  |  |  |  |  |  |
| Prostate cancer deaths | Main | 0 | 0 | Risk Ratio (M-H, Random, 95% CI) | Not estimable | - |
|  |  |  |  |  |  |  |
| Dichotomous markers of cancer risk | PSA >4ng/ml | 1 | 1622 | Risk Ratio (M-H, Random, 95% CI) | 1.13 [0.86, 1.50] | - |
|  |  |  |  |  |  |  |
| Continuous markers of cancer risk | PSA, ng/ml | 1 | 1622 | Mean Difference (IV, Random, 95% CI) | 0.10 [-0.03, 0.23] | - |

## Supplementary Table 6. High vs low ALA (secondary outcomes)

| **Outcome** | **Sensitivity Analysis (SA) or Subgroup** | **Studies** | **Participants** | **Statistical Method** | **Effect Estimate** | **I^2^, %** |
| --- | --- | --- | --- | --- | --- | --- |
| Quality of life | - | 0 | 0 | Mean Difference (IV, Random, 95% CI) | Not estimable | - |
|  |  |  |  |  |  |  |
| Adiposity | Weight, kg, ALA | 0 | 0 | Mean Difference (IV, Random, 95% CI) | Not estimable | - |
|  | BMI, kg/m2, ALA | 1 | 1260 | Mean Difference (IV, Random, 95% CI) | 0.15 [-0.03, 0.33] | - |
|  |  |  |  |  |  |  |
| Side effects | Drop outs due to side effects | 1 | 2433 | Risk Ratio (M-H, Random, 95% CI) | 0.89 [0.47, 1.65] | - |
|  | Any gastrointestinal side effect | 1 | 2433 | Risk Ratio (M-H, Random, 95% CI) | 0.93 [0.38, 2.28] | - |
| Dropouts |  | 0 | 0 | Risk Ratio (M-H, Random, 95% CI) | Not estimable | - |

## Supplementary Table 7. High vs low omega-6 (primary outcomes)

| **Outcome** | **Sensitivity Analysis (SA) or Subgroup** | **Studies** | **Participants** | **Statistical Method** | **Effect Estimate** | **I^2^, %** |
| --- | --- | --- | --- | --- | --- | --- |
| Cancer diagnoses | Main | 6 | 4272 | Risk Ratio (M-H, Random, 95% CI) | 1.21 [0.96, 1.53] | 0 |
|  | dietary advice & supplemental foods | 4 | 3961 | Risk Ratio (M-H, Random, 95% CI) | 1.17 [0.80, 1.70] | 35 |
|  | GLA supplement | 2 | 311 | Risk Ratio (M-H, Random, 95% CI) | 1.35 [0.31, 5.98] | 0 |
|  | SA fixed effects M-H | 6 | 4272 | Risk Ratio (M-H, Fixed, 95% CI) | 1.20 [0.95, 1.51] | 0 |
|  | SA fixed effects Peto | 6 | 4272 | Peto Odds Ratio (Peto, Fixed, 95% CI) | 1.22 [0.94, 1.57] | 14 |
|  | SA low summary RoB | 1 | 689 | Risk Ratio (M-H, Random, 95% CI) | 0.33 [0.01, 7.99] | - |
|  | SA compliance | 4 | 3961 | Risk Ratio (M-H, Random, 95% CI) | 1.17 [0.80, 1.70] | 35 |
|  | SA n >100 | 6 | 4272 | Risk Ratio (M-H, Random, 95% CI) | 1.21 [0.96, 1.53] | 0 |
|  |  |  |  |  |  |  |
| Cancer deaths | Main | 4 | 3321 | Risk Ratio (M-H, Random, 95% CI) | 0.97 [0.51, 1.85] | 52 |
|  | SA fixed effects M-H | 4 | 3321 | Risk Ratio (M-H, Fixed, 95% CI) | 1.12 [0.77, 1.64] | 52 |
|  | SA fixed effects Peto | 4 | 3321 | Peto Odds Ratio (Peto, Fixed, 95% CI) | 1.14 [0.74, 1.78] | 58 |
|  | SA low summary RoB | 0 | 0 | Risk Ratio (M-H, Random, 95% CI) | Not estimable | - |
|  | SA compliance | 3 | 3272 | Risk Ratio (M-H, Random, 95% CI) | 0.94 [0.33, 2.66] | 58 |
|  | SA n >100 | 3 | 3272 | Risk Ratio (M-H, Random, 95% CI) | 0.94 [0.33, 2.66] | 58 |
|  |  |  |  |  |  |  |
| Breast cancer diagnoses | Main | 1 | 200 | Risk Ratio (M-H, Random, 95% CI) | 1.00 [0.14, 6.96] | - |
|  | SA fixed effects M-H | 1 | 200 | Risk Ratio (M-H, Fixed, 95% CI) | 1.00 [0.14, 6.96] | - |
|  | SA fixed effects Peto | 1 | 200 | Peto Odds Ratio (Peto, Fixed, 95% CI) | 1.00 [0.14, 7.21] | - |
|  | SA low summary RoB | 0 | 0 | Risk Ratio (M-H, Random, 95% CI) | Not estimable | - |
|  | SA compliance | 0 | 0 | Risk Ratio (M-H, Random, 95% CI) | Not estimable | - |
|  | SA n >100 | 1 | 200 | Risk Ratio (M-H, Random, 95% CI) | 1.00 [0.14, 6.96] | - |
|  |  |  |  |  |  |  |
| Breast cancer deaths | Main | 0 | 0 | Risk Ratio (M-H, Random, 95% CI) | Not estimable | - |
|  |  |  |  |  |  |  |
| Prostate cancer diagnoses | Main | 1 | 2033 | Risk Ratio (M-H, Random, 95% CI) | 2.24 [0.69, 7.26] | - |
|  | SA fixed effects M-H | 1 | 2033 | Risk Ratio (M-H, Fixed, 95% CI) | 2.24 [0.69, 7.26] | - |
|  | SA fixed effects Peto | 1 | 2033 | Peto Odds Ratio (Peto, Fixed, 95% CI) | 2.16 [0.73, 6.43] | - |
|  | SA low summary RoB | 0 | 0 | Risk Ratio (M-H, Random, 95% CI) | Not estimable | - |
|  | SA compliance | 1 | 2033 | Risk Ratio (M-H, Random, 95% CI) | 2.24 [0.69, 7.26] | - |
|  | SA n >100 | 1 | 2033 | Risk Ratio (M-H, Random, 95% CI) | 2.24 [0.69, 7.26] | - |
|  |  |  |  |  |  |  |
| Prostate cancer deaths |  | 0 | 0 | Risk Ratio (M-H, Random, 95% CI) | Not estimable | - |
| Dichotomous markers of cancer risk |  | 0 | 0 | Mean Difference (IV, Random, 95% CI) | Not estimable | - |
| Continuous markers cancer risk | Breast density | 0 | 0 | Mean Difference (IV, Random, 95% CI) | Not estimable | - |
|  | PSA | 0 | 0 | Mean Difference (IV, Random, 95% CI) | Not estimable | - |

## Supplementary Table 8. High vs low omega-6 (secondary outcomes)

| **Outcome** | **Sensitivity Analysis (SA) or Subgroup** | **Studies** | **Participants** | **Statistical Method** | **Effect Estimate** | **I^2^, %** |
| --- | --- | --- | --- | --- | --- | --- |
| Quality of life |  | 0 | 0 | Mean Difference (IV, Random, 95% CI) | Not estimable | - |
| Adiposity | Weight, kg | 1 | 177 | Mean Difference (IV, Random, 95% CI) | Not estimable | - |
|  | BMI, kg/m^2^ | 0 | 0 | Mean Difference (IV, Random, 95% CI) | Not estimable | - |
| Side effects |  | 0 | 0 | Risk Ratio (M-H, Random, 95% CI) | Not estimable | - |
| Drop outs |  | 2 | 311 | Risk Ratio (M-H, Random, 95% CI) | 0.70 [0.40, 1.22] | 0 |

## Supplementary Table 9. GRADE table: summary of findings of effects of omega-6 fats on cancers

| **High compared to low omega 6 for cancer outcomes** | | | | | | |
| --- | --- | --- | --- | --- | --- | --- |
| **Patient or population**: adults, **Setting**: community, **Intervention**: Higher omega-6 intake, **Comparison**: low omega 6 intake | | | | | | |
| Outcomes | **Anticipated absolute effects^*^** (95% CI) | | Relative effect (95% CI) | № of participants  (studies) | Certainty of the evidence (GRADE) | Comments |
|  | **Risk with low omega 6 (primary outcomes)** | **Risk with High** |  |  |  |  |
| Cancer diagnoses | 56 per 1,000 | **68 per 1,000** (54 to 86) | **RR 1.21** (0.96 to 1.53) | 4272 (6 RCTs) | ⨁◯◯◯ VERY LOW ^a,b^ | The effect of increasing omega-6 on cancer diagnosis is unclear as the evidence is of very low quality. |
| Cancer deaths | 26 per 1,000 | **25 per 1,000** (13 to 48) | **RR 0.97** (0.51 to 1.85) | 3321 (4 RCTs) | ⨁◯◯◯ VERY LOW ^c,d,e^ | The effect of omega-6 on cancer deaths is unclear as the evidence is of very low quality. |
| Breast cancer diagnoses | 20 per 1,000 | **20 per 1,000** (3 to 139) | **RR 1.00** (0.14 to 6.96) | 200 (1 RCT) | ⨁◯◯◯ VERY LOW ^c,f,g^ | The effect of omega-6 on breast cancer diagnoses is unclear as the evidence is of very low quality. |
| Breast cancer deaths | not pooled | not pooled | not pooled | (0 RCTs) | - | We found no trials for this comparison |
| Prostate cancer diagnosis | 4 per 1,000 | **9 per 1,000** (3 to 29) | **RR 2.24** (0.69 to 7.26) | 2033 (1 RCT) | ⨁◯◯◯ VERY LOW ^c,e,f^ | The effect of omega-6 on risk of prostate cancer diagnosis is unclear as the evidence is of very low quality. |
| Prostate cancer death | 0 per 1,000 | **0 per 1,000** (0 to 0) | not estimable | (0 RCTs) | - | We found no trials assessing this effect. |
| ***The risk in the intervention group** (and its 95% confidence interval) is based on the assumed risk in the comparison group and the **relative effect** of the intervention (and its 95% CI).  **CI:** Confidence interval; **RR:** Risk ratio; **OR:** Odds ratio; **MD:** Mean difference | | | | | | |
| **GRADE Working Group grades of evidence** **High certainty:** We are very confident that the true effect lies close to that of the estimate of the effect **Moderate certainty:** We are moderately confident in the effect estimate: The true effect is likely to be close to the estimate of the effect, but there is a possibility that it is substantially different **Low certainty:** Our confidence in the effect estimate is limited: The true effect may be substantially different from the estimate of the effect **Very low certainty:** We have very little confidence in the effect estimate: The true effect is likely to be substantially different from the estimate of effect | | | | | | |

#### Explanations

a. Risk of bias: limiting analysis to trials at low summary risk of bias moves effect from harm to benefit (in the single remaining trial). Downgraded twice.

b. Imprecision: 95% CI includes harm and also no effect. Downgraded once.

c. Risk of bias: None of the included trials were at low summary risk of bias. Downgraded once.

d. Inconsistency: I2 was >50% but less than 60%. Effects differ according to analysis type in sensitivity analyses. Downgraded once.

e. Imprecision: 95% CI includes both benefits and harms. Downgraded once.

f. Indirectness: Only one trial assessed this outcome. Downgraded once.

g. Imprecision: 95% includes both important benefits and harms. Downgraded twice.

## Supplementary Table 10. High vs low total PUFA (primary outcomes)

| **Outcome** | **Sensitivity Analysis (SA) or Subgroup** | **Studies** | **Participants** | **Statistical Method** | **Effect Estimate** | **I^2^, %** | **p-value*** |
| --- | --- | --- | --- | --- | --- | --- | --- |
| Cancer diagnoses | Main | 8 | 9428 | Risk Ratio (M-H, Random, 95% CI) | 1.19 [0.99, 1.42] | 0 | - |
|  | SA fixed effects M-H | 8 | 9428 | Risk Ratio (M-H, Fixed, 95% CI) | 1.18 [0.98, 1.41] | 0 | - |
|  | SA fixed effects Peto | 8 | 9428 | Peto Odds Ratio (Peto, Fixed, 95% CI) | 1.19 [0.98, 1.45] | 0 | - |
|  | SA low summary risk of bias | 3 | 3262 | Risk Ratio (M-H, Random, 95% CI) | 1.08 [0.78, 1.51] | 0 | - |
|  | SA by compliance | 6 | 4230 | Risk Ratio (M-H, Random, 95% CI) | 1.20 [0.94, 1.54] | 5 | - |
|  | SA n >100 | 8 | 9428 | Risk Ratio (M-H, Random, 95% CI) | 1.19 [0.99, 1.42] | 0 | - |
|  | Duration: 1 to <2 years | 2 | 825 | Risk Ratio (M-H, Random, 95% CI) | 0.41 [0.06, 2.79] | 0 | 0.54 |
|  | Duration: 2 to <4 years | 2 | 2166 | Risk Ratio (M-H, Random, 95% CI) | 1.15 [0.86, 1.53] | 0 |  |
|  | Duration: 4+ years | 4 | 6437 | Risk Ratio (M-H, Random, 95% CI) | 1.22 [0.89, 1.69] | 27 |  |
|  | Dose of PUFA: <0.5%E | 0 | 0 | Risk Ratio (M-H, Random, 95% CI) | Not estimable | - | 0.89 |
|  | Dose of PUFA: 0.5 to <1.0%E | 1 | 136 | Risk Ratio (M-H, Random, 95% CI) | 0.47 [0.04, 5.08] | - |  |
|  | Dose of PUFA: 1.0 to <2.0%E | 1 | 2437 | Risk Ratio (M-H, Random, 95% CI) | 1.12 [0.80, 1.56] | - |  |
|  | Dose of PUFA: 2.0 to <5.0%E | 2 | 2166 | Risk Ratio (M-H, Random, 95% CI) | 1.15 [0.86, 1.53] | 0 |  |
|  | Dose of PUFA: ≥5.0%E | 3 | 1928 | Risk Ratio (M-H, Random, 95% CI) | 0.80 [0.24, 2.64] | 52 |  |
|  | Dose of PUFA: unclear | 1 | 2761 | Risk Ratio (M-H, Random, 95% CI) | 1.44 [0.57, 3.63] | - |  |
|  | PUFA replacing MUFA | 1 | 2761 | Risk Ratio (M-H, Random, 95% CI) | 1.44 [0.57, 3.63] | - | 0.50 |
|  | PUFA replacing mixed fats | 3 | 3115 | Risk Ratio (M-H, Random, 95% CI) | 0.81 [0.35, 1.85] | 29 |  |
|  | PUFA replacing SFA | 1 | 846 | Risk Ratio (M-H, Random, 95% CI) | 1.49 [1.01, 2.20] | - |  |
|  | PUFA replacing CHO | 3 | 2706 | Risk Ratio (M-H, Random, 95% CI) | 1.13 [0.83, 1.53] | 0 |  |
|  | Low risk - usual population | 6 | 6858 | Risk Ratio (M-H, Random, 95% CI) | 1.21 [0.93, 1.56] | 7 | 0.80 |
|  | Moderate risk - CA risk factors | 0 | 0 | Risk Ratio (M-H, Random, 95% CI) | Not estimable | - |  |
|  | High risk - previous cancer | 2 | 2570 | Risk Ratio (M-H, Random, 95% CI) | 1.14 [0.84, 1.56] | 0 |  |
|  | Mean age <50 years | 1 | 689 | Risk Ratio (M-H, Random, 95% CI) | 0.33 [0.01, 7.99] | - | 0.27 |
|  | Mean age 50- <65 | 5 | 5132 | Risk Ratio (M-H, Random, 95% CI) | 1.10 [0.89, 1.36] | 0 |  |
|  | Mean age 65+ | 2 | 3607 | Risk Ratio (M-H, Random, 95% CI) | 1.48 [1.04, 2.12] | 0 |  |
|  | Men & women mixed | 3 | 3030 | Risk Ratio (M-H, Random, 95% CI) | 1.28 [0.71, 2.30] | 0 | 0.92 |
|  | Men only | 4 | 3961 | Risk Ratio (M-H, Random, 95% CI) | 1.17 [0.80, 1.70] | 35 |  |
|  | Women only | 1 | 2437 | Risk Ratio (M-H, Random, 95% CI) | 1.12 [0.80, 1.56] | - |  |
|  |  |  |  |  |  |  |  |
| Cancer deaths | Main | 4 | 3408 | Risk Ratio (M-H, Random, 95% CI) | \| 1.10 [0.48, 2.49] \| \| --- \| | 37 | - |
|  | SA fixed effects M-H | 4 | 3408 | Risk Ratio (M-H, Fixed, 95% CI) | \| 1.27 [0.81, 1.99] \| \| --- \| | 37 | - |
|  | SA fixed effects Peto | 4 | 3408 | Peto Odds Ratio (Peto, Fixed, 95% CI) | 1.28 [0.80, 2.05] | 52 | - |
|  | SA low summary risk of bias | 1 | 136 | Risk Ratio (M-H, Random, 95% CI) | \| 1.89 [0.18, 20.31] \| \| --- \| | - | - |
|  | SA compliance | 4 | 3408 | Risk Ratio (M-H, Random, 95% CI) | 1.10 [0.48, 2.49] | 37 | - |
|  | SA n >100 | 4 | 3408 | Risk Ratio (M-H, Random, 95% CI) | 1.10 [0.48, 2.49] | 37 | - |
|  |  |  |  |  |  |  |  |
| Breast cancer diagnoses | Main | 2 | 5198 | Risk Ratio (M-H, Random, 95% CI) | 1.11 [0.71, 1.73] | 0 | - |
|  | SA fixed effects M-H | 2 | 5198 | Risk Ratio (M-H, Fixed, 95% CI) | 1.11 [0.71, 1.73] | 0 | - |
|  | SA fixed effects Peto | 2 | 5198 | Peto Odds Ratio (Peto, Fixed, 95% CI) | 1.11 [0.71, 1.75] | 0 | - |
|  | SA low summary risk of bias | 1 | 2437 | Risk Ratio (M-H, Random, 95% CI) | 1.03 [0.62, 1.71] | - | - |
|  | SA compliance | 0 | 0 | Risk Ratio (M-H, Random, 95% CI) | \| Not estimable \| \| --- \| | - | - |
|  | SA n >100 | 2 | 5198 | Risk Ratio (M-H, Random, 95% CI) | 1.11 [0.71, 1.73] | 0 | - |
|  |  |  |  |  |  |  |  |
| Breast cancer deaths | Main | 0 | 0 | Risk Ratio (M-H, Random, 95% CI) | Not estimable | - | - |
|  |  |  |  |  |  |  |  |
| Prostate cancer diagnoses | Main | 2 | 2879 | Risk Ratio (M-H, Random, 95% CI) | 1.64 [0.80, 3.36] | 0 | - |
|  | SA fixed effects M-H | 2 | 2879 | Risk Ratio (M-H, Fixed, 95% CI) | 1.66 [0.82, 3.38] | 0 | - |
|  | SA fixed effects Peto | 2 | 2879 | Peto Odds Ratio (Peto, Fixed, 95% CI) | 1.65 [0.82, 3.33] | 0 | - |
|  | SA low summary risk of bias | 0 | 0 | Risk Ratio (M-H, Random, 95% CI) | Not estimable | - | - |
|  | SA compliance | 2 | 2879 | Risk Ratio (M-H, Random, 95% CI) | 1.64 [0.80, 3.36] | 0 | - |
|  | SA n >100 | 2 | 2879 | Risk Ratio (M-H, Random, 95% CI) | 1.64 [0.80, 3.36] | 0 | - |
|  |  |  |  |  |  |  |  |
| Prostate cancer deaths | Main | 0 | 0 | Risk Ratio (M-H, Random, 95% CI) | Not estimable | - | - |
|  |  |  |  |  |  |  |  |
| Dichotomous markers of cancer risk |  | 0 | 0 | Risk Ratio (M-H, Random, 95% CI) | Not estimable | - | - |
|  |  |  |  |  |  |  |  |
| Continuous measures of cancer risk | Breast density | 0 | 0 | Mean Difference (IV, Random, 95% CI) | Not estimable | - | - |
|  | PSA | 0 | 0 | Mean Difference (IV, Random, 95% CI) | Not estimable | - | - |

## Supplementary Table 11. High vs low total PUFA (secondary outcomes)

| **Outcome** | **Sensitivity Analysis (SA) or Subgroup** | **Studies** | **Participants** | **Statistical Method** | **Effect Estimate** | **I^2^, %** |
| --- | --- | --- | --- | --- | --- | --- |
| Quality of life | Main | 0 | 0 | Mean Difference (IV, Random, 95% CI) | Not estimable | - |
|  |  |  |  |  |  |  |
| Adiposity | Weight, kg | 2 | 3800 | Mean Difference (IV, Random, 95% CI) | 0.37 [-0.05, 0.78] | 0 |
|  | BMI, kg/m^2^ | 1 | 320 | Mean Difference (IV, Random, 95% CI) | 0.01 [-0.30, 0.31] | 0 |
|  | Waist circumference, cm | 1 | 331 | Mean Difference (IV, Random, 95% CI) | 0.31 [-0.80, 1.43] | 0 |
|  |  |  |  |  |  |  |
| Side effects | Drop outs due to side effects | 0 | 0 | Risk Ratio (M-H, Random, 95% CI) | Not estimable | - |
|  | Bleeding | 0 | 0 | Risk Ratio (M-H, Random, 95% CI) | Not estimable | - |
| Drop outs |  | 0 | 0 | Odds Ratio (M-H, Fixed, 95% CI) | Not estimable | - |

## Supplementary Table 12. GRADE table: summary of findings of effects of total PUFA on cancers

| **High compared to low total PUFA for cancers** | | | | | | |
| --- | --- | --- | --- | --- | --- | --- |
| **Patient or population**: adults, **Setting**: community, **Intervention**: Higher total PUFA, **Comparison**: low total PUFA | | | | | | |
| Outcomes | **Anticipated absolute effects^*^** (95% CI) | | Relative effect (95% CI) | № of participants  (studies) | Certainty of the evidence (GRADE) | Comments |
|  | **Risk with low total PUFA (primary outcomes)** | **Risk with High** |  |  |  |  |
| Cancer diagnoses | 41 per 1,000 | **49 per 1,000** (41 to 58) | **RR 1.19** (0.99 to 1.42) | 9428 (8 RCTs) | ⨁⨁◯◯ LOW ^a,b,c^ | Increasing total PUFA may increase risk of diagnosis of any cancer. |
| Cancer deaths | 19 per 1,000 | **21 per 1,000** (9 to 47) | **RR 1.10** (0.48 to 2.49) | 3408 (4 RCTs) | ⨁⨁◯◯ LOW ^d^ | Increasing total PUFA may increase the risk of cancer death. |
| Breast cancer diagnoses | 13 per 1,000 | **14 per 1,000** (9 to 23) | **RR 1.11** (0.71 to 1.73) | 5198 (2 RCTs) | ⨁◯◯◯ VERY LOW ^e,f^ | The effect of increasing total PUFA on risk of breast cancer diagnosis is unclear as the evidence is of very low quality. |
| Breast cancer deaths | not pooled | not pooled | not pooled | (0 RCTs) | - | We found no trials assessing effects of total PUFA on breast cancer death. |
| Prostate cancer diagnoses | 8 per 1,000 | **14 per 1,000** (7 to 28) | **RR 1.64** (0.80 to 3.36) | 2879 (2 RCTs) | ⨁◯◯◯ VERY LOW ^f,g^ | The effect of increasing total PUFA on risk of prostate cancer diagnosis is unclear as the evidence is of very low quality. |
| Prostate cancer deaths | not pooled | not pooled | not pooled | (0 RCTs) | - | We found no trials assessing this outcome. |
| ***The risk in the intervention group** (and its 95% confidence interval) is based on the assumed risk in the comparison group and the **relative effect** of the intervention (and its 95% CI).  **CI:** Confidence interval; **RR:** Risk ratio; **MD:** Mean difference | | | | | | |
| **GRADE Working Group grades of evidence** **High certainty:** We are very confident that the true effect lies close to that of the estimate of the effect **Moderate certainty:** We are moderately confident in the effect estimate: The true effect is likely to be close to the estimate of the effect, but there is a possibility that it is substantially different **Low certainty:** Our confidence in the effect estimate is limited: The true effect may be substantially different from the estimate of the effect **Very low certainty:** We have very little confidence in the effect estimate: The true effect is likely to be substantially different from the estimate of effect | | | | | | |

#### Explanations

a. Risk of bias: Limiting to the 3 trials at low summary risk of bias moved the RR into "no effect" (RR 1.08). Downgraded once.

b. Imprecision: 95% CI includes no effect as well as harm. Downgraded once.

c. Publication bias: funnel plot suggests that if missing small studies were added into the meta-analysis it would increase RR. Not downgraded.

d. Imprecision: 95% CI includes important benefit as well as harm. Downgraded twice.

e. Risk of bias: Limiting to the single trial at low summary risk of bias moved the RR into "no effect" (RR 1.03). Downgraded once.

f. Imprecision: 95% CI includes important benefits and harms. Downgraded twice.

g. Risk of bias: no included trial was at low summary risk of bias. Downgraded once.

# References

1. Burr ML, Ashfield-Watt PA, Dunstan FD, Fehily AM, Breay P, Ashton T, et al. Lack of benefit of dietary advice to men with angina: results of a controlled trial. Eur J Clin Nutr. 2003;57(2):193-200.

2. Abdelhamid AS, Brown TJ, Brainard JS, Biswas P, Thorpe GC, Moore HJ, et al. Omega-3 fatty acids for the primary and secondary prevention of cardiovascular disease. Cochrane Database Syst Rev. 2018;11:CD003177. DOI: 10.1002/14651858.CD003177.pub4

3. Abdelhamid AS, Martin N, Bridges C, Brainard JS, Wang X, Brown TJ, et al. Polyunsaturated fatty acids for the primary and secondary prevention of cardiovascular disease. Cochrane Database Syst Rev. 2018;11:CD012345. DOI: 10.1002/14651858.CD012345.pub3

4. Hooper L, Al-Khudairy L, Abdelhamid AS, Rees K, Brainard JS, Brown TJ, et al. Omega-6 fats for the primary and secondary prevention of cardiovascular disease. Cochrane Database Syst Rev. 2018;11:CD011094. DOI: 10.1002/14651858.CD011094.pub4

5. Brouwer IA, Geleijnse JM, Klaasen VM, Smit LA, Giltay EJ, de Goede J, et al. Effect of alpha linolenic acid supplementation on serum prostate specific antigen (PSA): results from the alpha omega trial. PLoS ONE. 2013;8(12):e81519-e.

6. Giltay EJ, Geleijnse JM, Heijboer AC, de Goede J, Oude Griep LM, Blankenstein MA, et al. No effects of n-3 fatty acid supplementation on serum total testosterone levels in older men: the Alpha Omega Trial. Int J Androl. 2012;35(5):680-7.

7. Kromhout D. Alpha-Omega Trial Research Plan <https://www.alphaomegacohort.org/trial/2008> [

8. Kromhout D, Giltay EJ, Geleijnse JM, Alpha Omega Trial Group. n-3 fatty acids and cardiovascular events after myocardial infarction. N Engl J Med. 2010;363(21):2015-26.

9. Bonds DE, Harrington M, Worrall BB, Bertoni AG, Eaton CB, Writing Group for the Areds Research Group, et al. Effect of long-chain omega-3 fatty acids and lutein + zeaxanthin supplements on cardiovascular outcomes: results of the Age-Related Eye Disease Study 2 (AREDS2) randomized clinical trial. JAMA Intern Med. 2014;174(5):763-71.

10. Age-Related Eye Disease S. Lutein + zeaxanthin and omega-3 fatty acids for age-related macular degeneration: the Age-Related Eye Disease Study 2 (AREDS2) randomized clinical trial. JAMA. 2013;309(19):2005-15.

11. AREDS Research Group, Chew EY, Clemons T, SanGiovanni JP, Danis R, Domalpally A, et al. The Age-Related Eye Disease Study 2 (AREDS2): study design and baseline characteristics (AREDS2 report number 1). Ophthalmology. 2012;119(11):2282-9.

12. ASCEND Study Collaborative Group. Effects of n−3 Fatty Acid Supplements in Diabetes Mellitus. N Engl J Med. 2018;379(16):1540-50. 10.1056/NEJMoa1804989

13. Bowman L, Aung T, Haynes R, Armitage J. ASCEND: Design and baseline characteristics of a large randomised trial in diabetes. Diabetes. 2012;61:A556-A7.

14. Berson EL, Rosner B, Sandberg MA, Weigel-DiFranco C, Moser A, Brockhurst RJ, et al. Clinical trial of docosahexaenoic acid in patients with retinitis pigmentosa receiving vitamin A treatment. Arch Ophthalmol. 2004;122(9):1297-305.

15. Berson EL, Rosner B, Sandberg MA, Weigel-DiFranco C, Moser A, Brockhurst RJ, et al. Further evaluation of docosahexaenoic acid in patients with retinitis pigmentosa receiving vitamin A treatment: subgroup analyses. Arch Ophthalmol. 2004;122(9):1306-14.

16. Black HS, Herd JA, Goldberg LH, Wolf-Je J, Thornby JI, Rosen T, et al. Effect of a low-fat diet on the incidence of actinic keratosis. N Engl J Med. 1994;330(18):1272-5.

17. Black HS, Thornby JI, Wolf-Je J, Goldberg LH, Herd JA, Rosen T, et al. Evidence that a low-fat diet reduces the occurrence of non-melanoma skin cancer. Int J Cancer. 1995;62(2):165-9.

18. Burr ML, Fehily AM, Gilbert JF, Rogers S, Holliday RM, Sweetnam PM, et al. Effects of changes in fat, fish, and fibre intakes on death and myocardial reinfarction: diet and reinfarction trial (DART). Lancet. 1989;2(8666):757-61.

19. Burr ML, Fehily AM, Rogers S, Welsby E, King S, Sandham S. Diet and reinfarction trial (DART): design, recruitment, and compliance. Eur Heart J. 1989;10(6):558-67.

20. Fehily AM, Vaughan-Williams E, Shiels K, Williams AH, Horner M, Bingham G, et al. The effect of dietary advice on nutrient intakes: Evidence from the diet and reinfarction trial (DART). J Hum Nutr Diet. 1989;2(4):225-35.

21. Einvik G, Ekeberg O, Lavik JG, Ellingsen I, Klemsdal TO, Hjerkinn EM. The influence of long-term awareness of hyperlipidemia and of 3 years of dietary counseling on depression, anxiety, and quality of life. J Psychosom Res. 2010;68(6):567-72.

22. Einvik G, Klemsdal TO, Sandvik L, Hjerkinn EM. A randomized clinical trial on n-3 polyunsaturated fatty acids supplementation and all-cause mortality in elderly men at high cardiovascular risk. Eur J Cardiovasc Prev Rehabil. 2010;17(5):588-92.

23. Sanyal AJ, Abdelmalek MF, Suzuki A, Cummings OW, Chojkier M, Group E. No significant effects of ethyl-eicosapentanoic acid on histologic features of nonalcoholic steatohepatitis in a phase 2 trial. Gastroenterology. 2014;147(2):377-84.

24. Feagan BG, Sandborn WJ, Mittmann U, Bar-Meir S, D'Haens G, Bradette M, et al. Omega-3 free fatty acids for the maintenance of remission in Crohn disease: the EPIC Randomized Controlled Trials. JAMA. 2008;299(14):1690-7.

25. Hill CL, March LM, Aitken D, Lester SE, Battersby R, Hynes K, et al. Fish oil in knee osteoarthritis: a randomised clinical trial of low dose versus high dose. Ann Rheum Dis. 2016;75(1):23-9.

26. Gissi-Hf Investigators, Tavazzi L, Maggioni AP, Marchioli R, Barlera S, Franzosi MG, et al. Effect of n-3 polyunsaturated fatty acids in patients with chronic heart failure (the GISSI-HF trial): a randomised, double-blind, placebo-controlled trial. Lancet. 2008;372(9645):1223-30.

27. Tavazzi L, Tognoni G, Franzosi MG, Latini R, Maggioni AP, Marchioli R, et al. Rationale and design of the GISSI heart failure trial: a large trial to assess the effects of n-3 polyunsaturated fatty acids and rosuvastatin in symptomatic congestive heart failure. Eur J Heart Fail. 2004;6(5):635-41.

28. Gissi-Prevenzione Investigators. Dietary supplementation with n-3 polyunsaturated fatty acids and vitamin E after myocardial infarction: results of the GISSI-Prevenzione trial. Lancet. 1999;354:447-55.

29. Keen H, Payan J, Allawi J, Walker J, Jamal GA, Weir AI, et al. Treatment of diabetic neuropathy with gamma-linolenic acid. The gamma-Linolenic Acid Multicenter Trial Group. Diabetes Care. 1993;16(1):8-15.

30. Sacks FM, Stone PH, Gibson CM, Silverman DI, Rosner B, Pasternak RC. Controlled trial of fish oil for regression of human coronary atherosclerosis. HARP Research Group. J Am Coll Cardiol. 1995;25(7):1492-8.

31. Higashihara E, Itomura M, Terachi T, Matsuda T, Kawakita M, Kameyama S, et al. Effects of eicosapentaenoic acid on biochemical failure after radical prostatectomy for prostate cancer. In Vivo. 2010;24(4):561-5.

32. Huang YC, Jessup JM, Forse RA, Flickner S, Pleskow D, Anastopoulos HT, et al. N-3 fatty acids decrease colonic epithelial cell proliferation in high-risk bowel mucosa. Lipids. 1996;31:S313-S7.

33. Yokoyama M, Origasa H, Matsuzaki M, Matsuzawa Y, Saito Y, Ishikawa Y, et al. Effects of eicosapentaenoic acid on major coronary events in hypercholesterolaemic patients (JELIS): a randomised open-label, blinded endpoint analysis. Lancet. 2007;369(9567):1090-8.

34. Ley SJ, Metcalf PA, Scragg RKR, Swinburn BA. Long-term effects of a reduced fat diet intervention on cardiovascular disease risk factors in individuals with glucose intolerance. Diabetes Research and Clinical Practice. 2004;63:103-12.

35. Swinburn BA, Metcalf PA, Ley SJ. Long-term (5-year) effects of a reduced-fat diet intervention in individuals with glucose intolerance. Diabetes Care. 2001;24(4):619-24.

36. Macsai MS. The role of omega-3 dietary supplementation in blepharitis and meibomian gland dysfunction (an AOS thesis). Trans Am Ophthalmol Soc. 2008;106:336-56.

37. Mansel RE, Gateley CA, Harrison BJ, Melhuish J, Sheridan W, Pye JK, et al. Effects and tolerability of n-6 essential fatty acid supplementation in patients with recurrent breast cysts -- a randomized double-blind placebo-controlled trial. Journal of Nutritional Medicine. 1990;1(3):195-.

38. Mansel RE, Harrison BJ, Melhuish J, Sheridan W, Pye JK, Pritchard G, et al. A randomized trial of dietary intervention with essential fatty acids in patients with categorized cysts. Ann N Y Acad Sci. 1990;586:288-94.

39. Mansel RE, Pye JK, Hughes LE. Effects of essential fatty acids on cyclical mastalgia and noncyclical breat disorders. Omega-6 Essential Fatty Acids: Pathophysiology and Roles in Clinical Medicine. 1990:557-66.

40. McIllmurray MB, Turkie W. Controlled trial of gamma linolenic acid in Duke's C colorectal cancer. British Medical Journal (Clinical research ed). 1987;294(6582):1260 (correction BMJ 987;295(6596):475)-1260 (correction BMJ 987;295(6596):475).

41. Mita T, Watada H, Ogihara T, Nomiyama T, Ogawa O, Kinoshita J, et al. Eicosapentaenoic acid reduces the progression of carotid intima-media thickness in patients with type 2 diabetes. Atherosclerosis. 2007;191(1):162-7.

42. MRC. Controlled trial of soya-bean oil in myocardial infarction. Lancet. 1968;2(570):693-9.

43. Ederer F, Leren P, Turpeinen O, Frantz Id Jr. Cancer among men on cholesterol lowering diets: experience of five clinical trials. Lancet. 1971;2:203-6.

44. Heady JA. Are PUFA harmful? BMJ (Clinical Research Ed). 1974;1:115-6.

45. NDHS Research Group. The National Diet-Heart Study final report. Circulation. 1968;37(II):1-428.

46. Nilsen DW, Albrektsen G, Landmark K, Moen S, Aarsland T, Woie L. Effects of a high-dose concentrate of n-3 fatty acids or corn oil introduced early after an acute myocardial infarction on serum triacylglycerol and HDL cholesterol. Am J Clin Nutr. 2001;74(1):50-6.

47. Rauch B, Schiele R, Schneider S, Diller F, Victor N, Gohlke H, et al. OMEGA, a randomized, placebo-controlled trial to test the effect of highly purified omega-3 fatty acids on top of modern guideline-adjusted therapy after myocardial infarction. Circulation. 2010;122(21):2152-9.

48. Rauch B, Schiele R, Schneider S, Gohlke H, Diller F, Gottwik M, et al. Highly purified omega-3 fatty acids for secondary prevention of sudden cardiac death after myocardial infarction-aims and methods of the OMEGA-study. Cardiovascular Drugs Therapy. 2006;20(5):365-75.

49. Bordeleau L, Yakubovich N, Dagenais G, Rosenstock J, Ryden LE, Spinas G, et al. Cancer outcomes in patients with dysglycemia on basal insulin: results of the origin trial. Diabetes. 2013;62:A98.

50. Bordeleau L, Yakubovich N, Dagenais GR, Rosenstock J, Probstfield J, Chang Yu P, et al. The association of basal insulin glargine and/or n-3 fatty acids with incident cancers in patients with dysglycemia. Diabetes Care. 2014;37(5):1360-6.

51. Origin Trial Investigators, Bosch J, Gerstein HC, Dagenais GR, Diaz R, Dyal L, et al. n-3 fatty acids and cardiovascular outcomes in patients with dysglycemia. N Engl J Med. 2012;367(4):309-18.

52. Tatsuno I. Omega-3 polyunsaturated fatty acids and cardiovascular disease: an emphasis on omega-3-acid ethyl esters 90 for the treatment of hypertriglyceridemia. Expert Rev Cardiovasc Ther. 2014;12(11):1261-8.

53. Tatsuno I, Saito Y, Kudou K, Ootake J. Long-term safety and efficacy of TAK-085 in Japanese subjects with hypertriglyceridemia undergoing lifestyle modification: the omega-3 fatty acids randomized long-term (ORL) study. J Clin Lipidol. 2013;7(6):615-25.

54. Estruch R, Ros E, Salas-Salvadó J, Covas M, Corella D, Arós F et al. Retraction and republication: Primary prevention of cardiovascular disease with a Mediterranean diet. N Engl J Med 2013; 368:1279-90. N Engl J Med. 2018;378:25-.

55. Toledo E, Salas-Salvado J, Donat-Vargas C, Buil-Cosiales P, Estruch R, Ros E, et al. Mediterranean Diet and Invasive Breast Cancer Risk Among Women at High Cardiovascular Risk in the PREDIMED Trial: A Randomized Clinical Trial. JAMA Intern Med. 2015;175(11):1752-60.

56. Puri BK, Leavitt BR, Hayden MR, Ross CA, Rosenblatt A, Greenamyre JT, et al. Ethyl-EPA in Huntington disease: a double-blind, randomized, placebo-controlled trial. Neurology. 2005;65(2):286-92.

57. Raitt MH, Connor WE, Morris C, Kron J, Halperin B, Chugh SS, et al. Fish oil supplementation and risk of ventricular tachycardia and ventricular fibrillation in patients with implantable defibrillators: a randomized controlled trial. JAMA. 2005;293(23):2884-91.

58. Rischio, Prevenzione I. Efficacy of n-3 polyunsaturated fatty acids and feasibility of optimizing preventive strategies in patients at high cardiovascular risk: rationale, design and baseline characteristics of the Rischio and Prevenzione study, a large randomised trial in general practice. Trials. 2010;11:68.

59. Roncaglioni MC, Tombesi M, Avanzini F, Barlera S, Caimi V, Longoni P, et al. n-3 Fatty Acids in Patients with Multiple Cardiovascular Risk Factors. N Engl J Med. 2013;368(19):1800-8. 10.1056/NEJMoa1205409

60. Myrup B, Rossing P, Jensen T, Parving HH, Holmer G, Gram J, et al. Lack of effect of fish oil supplementation on coagulation and transcapillary escape rate of albumin in insulin-dependent diabetic patients with diabetic nephropathy. Scand J Clin Lab Invest. 2001;61(5):349-56.

61. Rossing P, Hansen BV, Nielsen FS, Myrup B, Holmer G, Parving HH. Fish oil in diabetic nephropathy. Diabetes Care. 1996;19(11):1214-9.

62. Sandhu N, Schetter SE, Liao J, Hartman TJ, Richie JP, McGinley J, et al. Influence of Obesity on Breast Density Reduction by Omega-3 Fatty Acids: Evidence from a Randomized Clinical Trial. Cancer Prev Res. 2016;9(4):275-82.

63. Signori C, DuBrock C, Richie JP, Prokopczyk B, Demers LM, Hamilton C, et al. Administration of omega-3 fatty acids and Raloxifene to women at high risk of breast cancer: interim feasibility and biomarkers analysis from a clinical trial. Eur J Clin Nutr. 2012;66(8):878-84.

64. Angerer P, Kothny W, Stork S, von Schacky C. Effect of dietary supplementation with omega-3 fatty acids on progression of atherosclerosis in carotid arteries. Cardiovascular Research. 2002;54(1):183-90.

65. von Schacky C, Angerer P, Kothny W, Theisen K, Mudra H. The effect of dietary omega-3 fatty acids on coronary atherosclerosis. A randomized, double-blind, placebo-controlled trial. Ann Intern Med. 1999;130(7):554-62.

66. von Schacky C, Baumann K, Angerer P. The effect of n-3 fatty acids on coronary atherosclerosis: results from SCIMO, an angiographic study, background and implications. Lipids. 2001;36 Suppl:S99-102.

67. Hull MA, Sprange K, Hepburn T, Tan W, Shafayat A, Rees CJ, et al. Eicosapentaenoic acid and aspirin, alone and in combination, for the prevention of colorectal adenomas (seAFOod Polyp Prevention trial): a multicentre, randomised, double-blind, placebo-controlled, 2&#x2008;&#xd7;&#x2008;2 factorial trial. The Lancet. 2018;392(10164):2583-94. 10.1016/S0140-6736(18)31775-6

68. Simon MS, Heilbrun LK, Boomer A, Kresge C, Depper J, Kim PN, et al. A randomised trial of a low-fat dietary intervention in women at high risk for breast cancer. Nutr Cancer. 1997;27(2):136-42.

69. Brouwer IA, Geelen A, Katan MB. n-3 Fatty acids, cardiac arrhythmia and fatal coronary heart disease. Prog Lipid Res. 2006;45(4):357-67.

70. Brouwer IA, Katan MB, Schouten EG, Camm AJ, Hauer RNW, Wever EFD, et al. Rationale and design of a clinical trial on n-3 fatty acids and cardiac arrhythmia (SOFA). Ann Nutr Metab. 2001;45(Suppl 1):79-.

71. Brouwer IA, Katan MB, Zock PL. Effects of n-3 fatty acids on arrhythmic events and mortality in the SOFA implantable cardioverter defibrillator trial. Am J Clin Nutr. 2006;84(6):1554.

72. Brouwer IA, Zock PL, Camm AJ, Bocker D, Hauer RN, Wever EF, et al. Effect of fish oil on ventricular tachyarrhythmia and death in patients with implantable cardioverter defibrillators: the Study on Omega-3 Fatty Acids and Ventricular Arrhythmia (SOFA) randomized trial. JAMA. 2006;295(22):2613-9.

73. Andreeva VA, Latarche C, Hercberg S, Briancon S, Galan P, Kesse-Guyot E. B vitamin and/or n-3 fatty acid supplementation and health-related quality of life: ancillary findings from the SU.FOL.OM3 randomized trial. PLoS ONE. 2014;9(1):e84844.

74. Andreeva VA, Touvier M, Kesse-Guyot E, Julia C, Galan P, Hercberg S. B vitamin and/or omega-3 fatty acid supplementation and cancer: ancillary findings from the supplementation with folate, vitamins B6 and B12, and/or omega-3 fatty acids (SU.FOL.OM3) randomized trial. Arch Intern Med. 2012;172(7):540-7.

75. Galan P, Briancon S, Blacher J, Czernichow S, Hercberg S. The SU.FOL.OM3 Study: a secondary prevention trial testing the impact of supplementation with folate and B-vitamins and/or Omega-3 PUFA on fatal and non fatal cardiovascular events, design, methods and participants characteristics. Trials. 2008;9:35.

76. Galan P, de Bree A, Mennen L, Potier de Courcy G, Preziozi P, Bertrais S, et al. Background and rationale of the SU.FOL.OM3 study: double-blind randomized placebo-controlled secondary prevention trial to test the impact of supplementation with folate, vitamin B6 and B12 and/or omega-3 fatty acids on the prevention of recurrent ischemic events in subjects with atherosclerosis in the coronary or cerebral arteries. J Nutr Health Aging. 2003;7(6):428-35.

77. Galan P, Kesse-Guyot E, Czernichow S, Briancon S, Blacher J, Hercberg S. Effects of B vitamins and omega 3 fatty acids on cardiovascular diseases: A randomised placebo controlled trial. Br Med J. 2011;342(7787):36.

78. Touvier M, Kesse-Guyot E, Andreeva VA, Fezeu L, Charnaux N, Sutton A, et al. Modulation of the association between plasma intercellular adhesion molecule-1 and cancer risk by n-3 PUFA intake: a nested case-control study. Am J Clin Nutr. 2012;95(4):944-50.

79. Tuttle KR, Shuler LA, Packard DP, Milton JE, Daratha KB, Bibus DM, et al. Comparison of low-fat versus Mediterranean-style dietary intervention after first myocardial infarction (from The Heart Institute of Spokane Diet Intervention and Evaluation Trial). Am J Cardiol. 2008;101(11):1523-30.

80. Dayton S, Pearce ML, Hashimoto S, Dixon WJ, Tomayasu U. A controlled clinical trial of a diet high in unsaturated fat in preventing complications of atherosclerosis. Circulation. 1969;15(1, Suppl 2):II-1-63.

81. Pearce ML, Dayton S. Incidence of cancer in men on a diet high in polyunsaturated fat. Lancet. 1971;1(7697):464-7.

82. Manson JE, Cook NR, Lee IM, Christen W, Bassuk SS, Mora S, et al. Marine n−3 Fatty Acids and Prevention of Cardiovascular Disease and Cancer. N Engl J Med. 2018. 10.1056/NEJMoa1811403

83. Huey L, Bitok E, Kazzi N, Sirirat R, Haddad Tabrizi S, Ros E, et al. Dietary compliance of walnut or no walnut intake in a 1-year randomized intervention trial among free-living elderly in the Walnuts and Healthy Aging Study (WAHA). FASEB Journal2016. p. 1157.10-.10.

84. Ros E, Rajaram S, Sala-Vila A, Serra-Mir M, Valls-Pedret C, Cofan M, et al. Effect of a 1-year walnut supplementation on blood lipids among older individuals: Findings from the walnuts and healthy aging (WAHA) study. FASEB Journal. 2016;30(Supp 1):293-4.

85. Rajaram S, Valls-Pedret C, Cofan M, Sabate J, Serra-Mir M, Perez-Heras AM, et al. The Walnuts and Healthy Aging Study (WAHA): Protocol for a Nutritional Intervention Trial with Walnuts on Brain Aging. Front Aging Neurosci. 2016;8:333-.

86. Chlebowski RT, Blackburn GL, Thomson CA, Nixon DW, Shapiro A, Hoy MK, et al. Dietary fat reduction and breast cancer outcome: interim efficacy results from the women's intervention nutrition study. JNCI Journal of the National Cancer Institute. 2006;98(24):1767-76.

87. Chlebowski RT, Rose DP, Buzzard IM, Blackburn GL, York M, Insull W, et al. Dietary fat reduction in adjuvant breast cancer therapy: current rationale and feasibility issues. Adjuvant The Cancer Journal. 1990;6:357-63.

88. Rose DP, Connolly JM, Chlebowski RT, Buzzard IM, Wynder EL. The effects of a low-fat dietary intervention and tamoxifen adjuvant therapy on the serum estrogen and sex hormone-binding globulin concentrations of postmenopausal breast cancer patients. Breast Cancer Res Treat. 1993;27(3):253-62.
